# Understanding and predicting animal movements and distributions in the Anthropocene

**DOI:** 10.1111/1365-2656.70040

**Published:** 2025-04-04

**Authors:** Sara Gomez, Holly M. English, Vanesa Bejarano Alegre, Paul G. Blackwell, Anna M. Bracken, Eloise Bray, Luke C. Evans, Jelaine L. Gan, W. James Grecian, Catherine Gutmann Roberts, Seth M. Harju, Pavla Hejcmanová, Lucie Lelotte, Benjamin Michael Marshall, Jason Matthiopoulos, AichiMkunde Josephat Mnenge, Bernardo Brandao Niebuhr, Zaida Ortega, Christopher J. Pollock, Jonathan R. Potts, Charlie J. G. Russell, Christian Rutz, Navinder J. Singh, Katherine F. Whyte, Luca Börger

**Affiliations:** ^1^ CEFE, Univ Montpellier, CNRS, EPHE, IRD Montpellier France; ^2^ School of Biology and Environmental Science University College Dublin Dublin Ireland; ^3^ Spatial Ecology and Conservation Lab (LEEC), Department of Biodiversity Institute of Biosciences, São Paulo State University‐UNESP Rio Claro São Paulo Brazil; ^4^ School of Mathematical and Physical Sciences University of Sheffield Sheffield UK; ^5^ School of Biodiversity, One Health and Veterinary Medicine University of Glasgow Glasgow UK; ^6^ School of Biological Sciences University of Reading Reading UK; ^7^ School of Natural and Environmental Sciences Newcastle University Newcastle upon Tyne UK; ^8^ University of the Philippines Quezon City Philippines; ^9^ Department of Geography Durham University Durham UK; ^10^ School of Biological and Marine Sciences University of Plymouth Plymouth UK; ^11^ Heron Ecological Kingston Idaho USA; ^12^ Faculty of Tropical AgriSciences Czech University of Life Sciences Prague Prague Czechia; ^13^ Department of Biology, Ecology and Evolution University of Liege Liege Belgium; ^14^ Biological and Environmental Sciences, Faculty of Natural Sciences University of Stirling Stirling UK; ^15^ Zoology Department Nelson Mandela University Port Elizabeth South Africa; ^16^ Zoological Society of London London UK; ^17^ Norwegian Institute for Nature Research Oslo Norway; ^18^ Department of Biodiversity and Environmental Management University of León León Spain; ^19^ UK Centre for Ecology & Hydrology Penicuik UK; ^20^ School of Environmental Sciences University of East Anglia Norwich UK; ^21^ British Trust for Ornithology UK; ^22^ Centre for Biological Diversity, School of Biology University of St Andrews St Andrews UK; ^23^ Department of Wildlife, Fish and Environmental Studies Swedish University of Agricultural Sciences Umeå Sweden; ^24^ Biomathematics and Statistics Scotland Edinburgh UK; ^25^ Department of Biosciences Swansea University Swansea UK

**Keywords:** biologging, conservation, human‐modified landscapes, modelling, movement ecology

## Abstract

1. Predicting animal movements and spatial distributions is crucial for our comprehension of ecological processes and provides key evidence for conserving and managing populations, species and ecosystems. Notwithstanding considerable progress in movement ecology in recent decades, developing robust predictions for rapidly changing environments remains challenging.
2. To accurately predict the effects of anthropogenic change, it is important to first identify the defining features of human‐modified environments and their consequences on the drivers of animal movement. We review and discuss these features within the movement ecology framework, describing relationships between external environment, internal state, navigation and motion capacity.
3. Developing robust predictions under novel situations requires models moving beyond purely correlative approaches to a dynamical systems perspective. This requires increased mechanistic modelling, using functional parameters derived from first principles of animal movement and decision‐making. Theory and empirical observations should be better integrated by using experimental approaches. Models should be fitted to new and historic data gathered across a wide range of contrasting environmental conditions. We need therefore a targeted and supervised approach to data collection, increasing the range of studied taxa and carefully considering issues of scale and bias, and mechanistic modelling. Thus, we caution against the indiscriminate non‐supervised use of citizen science data, AI and machine learning models.
4. We highlight the challenges and opportunities of incorporating movement predictions into management actions and policy. Rewilding and translocation schemes offer exciting opportunities to collect data from novel environments, enabling tests of model predictions across varied contexts and scales. Adaptive management frameworks in particular, based on a stepwise iterative process, including predictions and refinements, provide exciting opportunities of mutual benefit to movement ecology and conservation.
5. In conclusion, movement ecology is on the verge of transforming from a descriptive to a predictive science. This is a timely progression, given that robust predictions under rapidly changing environmental conditions are now more urgently needed than ever for evidence‐based management and policy decisions. Our key aim now is not to describe the existing data as well as possible, but rather to understand the underlying mechanisms and develop models with reliable predictive ability in novel situations.

## Graphical abstract

This review provides a framework to better understand and predict animal movements and distributions in human‐modified environments. Bridging the gap between movement models and management/conservation policy is challenging. Here, the recommendations presented highlight the exciting opportunities now available to advance the field of movement ecology into a more predictive science.

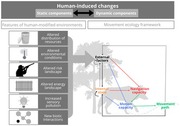

## 1 INTRODUCTION

Humans have impacted Earth's systems globally (Cowie et al., 2022), affecting both climate (Calvin et al., 2023) and biodiversity (Pereira et al., 2024; Venter et al., 2016), including species distributions (Chan et al., 2024), local ecosystem structure and functioning (Davoli et al., 2024), and eco‐evolutionary processes (Boughman et al., 2024). Collectively, these anthropogenic changes are pushing Earth's systems beyond sustainable function, with potentially catastrophic impacts for humanity (Richardson et al., 2023). We must urgently develop strategies for mitigating these adverse effects. As ecosystems are complex and interrelated, and increasingly changing to a novel or emerging state under human influence (Svenning, Buitenwerf, et al., 2024), they cannot be protected with localised measures (Harris et al., 2024; Svenning, Buitenwerf, et al., 2024). As such, an increased focus on underlying processes is required to safeguard global ecosystem functioning.

A key process to inform policy and management decisions is animal movement, as animals connect ecotopes and ecosystems, move vast amounts of biomass, genes and energy across the globe, and maintain functional ecosystems (Gable et al., 2023; Schmitz et al., 2023). Under global change, animals are increasingly exposed to new and unfamiliar conditions and novel ecosystems (Doherty et al., 2021; Hobbs et al., 2006), including human‐modified landscapes such as those created by forest conversion to agricultural or urban areas (Arroyo‐Rodríguez et al., 2020) and new species assemblages due to invasive species (Harris et al., 2024). Movement is a key behavioural response to environmental change (Tucker et al., 2023; Tuomainen & Candolin, 2011) and is critical in determining larger‐scale population‐level spatial patterns (Matthiopoulos, 2003) and biodiversity patterns (Schlägel et al., 2019). Individuals move in response to their internal state and navigational capacity, modulated by local environmental context, including anthropogenic activity (Nathan et al., 2008; Owen et al., 2017; Shaw, 2020). Together, these individual movement decisions scale up to shape species' spatial distributions. Individual and species‐level movements are hence intrinsically linked, each providing an alternative approach to understanding the drivers of wild animal populations under human pressures. For example, movement often provides a useful framework for biological questions concerning behaviour (i.e. a microscopic view), while animal distributions are more appropriate for understanding population‐based questions (i.e. a macroscopic view).

In recent decades, there has been a rapid increase in our ability to record, process and model animal movements (Hussey et al., 2015; Kays et al., 2015; Nathan et al., 2022) and behaviour (Williams et al., 2020). This has created exciting opportunities to develop predictive models of animal movements and distributions (e.g. Signer et al., 2024). In particular, predicting (changes in) animal movements would allow us to anticipate and mitigate human‐modified impacts. This is notoriously difficult to achieve, however, especially under novel conditions. We thus urgently need practical methodological frameworks to understand the drivers of animal movement and distributions in human‐modified landscapes, to improve policy and management decisions (Allen & Singh, 2016; Hays et al., 2019).

Here, we highlight the need to build a predictive framework of how animals move in changing environments. This should account for terrestrial, aerial, freshwater and marine species with global coverage, while acknowledging the high degree of context dependence (Ma et al., 2024). Specifically, we aim to: (1) define human‐modified environments and how they impact animal movements; (2) provide an overview of the current knowledge and challenges associated with predictive movement modelling; and (3) discuss approaches to incorporate predictions of animal movements and distributions into management actions and policy. We believe that improved predictions of animal movements and distributions in human‐modified environments will considerably enhance current strategies aiming to mitigate the biodiversity crisis.

## 2 HUMAN‐MODIFIED ENVIRONMENTS: DEFINING FEATURES, DRIVERS OF MOVEMENTS AND IMPACTS

Anthropogenic changes are profoundly affecting Earth's biosphere, leading to the emergence of novel, or highly altered, ecosystems. It is becoming increasingly important to understand and predict the dynamics of ecosystems under these unprecedented or novel conditions (Svenning, McGeoch, et al., 2024). Here, we present the defining features of human‐modified environments and their consequences as drivers of animal movement.

### 2.1 Defining ‘human‐modified environments’

Natural environments can change drastically as a result of natural disturbance events, such as wildfires, storms or earthquakes, affecting the diversity, structure and dynamics of ecological communities (Levin & Paine, 1974). Human pressures have been accelerating the rate of such changes, challenging the ability of wildlife to adapt. We define ‘human‐induced changes’ as environmental changes resulting from pressures created by human activities that often cause local and global impacts on natural environments (see the ‘human footprint framework’; Venter et al., 2016). These include changes in land use and cover, natural resource extraction, construction and sensory pollution. ‘Human‐modified environments’ are products of these human‐induced changes. It is essential to understand the features of natural and human‐induced changes, how they interact and how they relate to the temporal and spatial scales on which animals usually function. Human‐induced changes often exhibit distinctive temporal and spatial attributes compared with natural processes (e.g. higher intensity, increased frequency over time or broader spatial extents).

**FIGURE 1:**
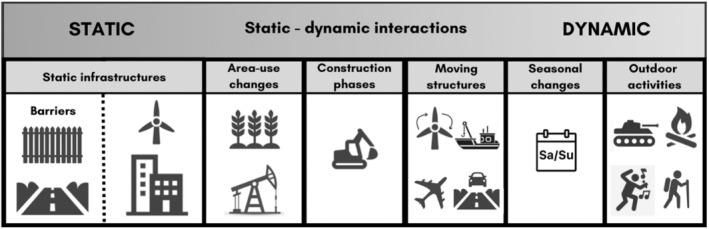
Static and dynamic components of human‐induced changes. Human‐induced changes can be categorised along a static–dynamic continuum where barriers and constructions can be defined as truly static and human outdoor activities can be defined as truly dynamic. In between, there are other human‐induced changes where static and dynamic disturbances interact (e.g. roads—see main text).

Different types of disturbance vary in their temporal traits (e.g. continuous versus discrete and acute versus chronic) and impacts on wildlife and ecosystems, differentially affecting not only the equilibrium states but also the transient community dynamics (Inamine et al., 2022). Previous work on the environmental impacts of human activity distinguished between relatively ‘static’ landscape modifications (e.g. roads and buildings) and the ‘dynamic’ movement of humans and their vehicles, and any associated by‐products (‘human mobility’) (Ellis‐Soto et al., 2023; Rutz, 2022b); see also earlier work on the pulse and press disturbance framework (Bender et al., 1984). Here, we expand this framing, exploring a continuum from static to dynamic disturbances that are relevant across time scales (see Figure 1). Static disturbances are changes to the structure of the landscape—such as buildings, transportation lines, fences and dams—which often have longer‐term impacts on the movements of individual animals, population dynamics and species distributions (Benítez‐López et al., 2010; Rutz, 2022b). Aside from infrastructure development, established area‐use changes caused by agriculture, mining, trawling and oil extraction can also be considered static, due to their complex structural impacts which are not easily or quickly reversed (Figure 1).

On the other hand, dynamic disturbances are temporary alterations; they can affect animals immediately or after response lags. Many are determined by the movements of humans and their vehicles across the environment, including the release of by‐products, such as light, noise and pollutants (‘human mobility’, as defined in Rutz 2022b), but more generally can include dynamic changes such as modified wind speeds and turbulences caused by wind turbines (‘wake effect’) or pulses of pollution caused by wastewater discharges. In addition, dynamic changes vary in intensity across time scales, and may be regular (e.g. increased human presence during holidays or the ‘weekend effect’) or unpredictable over time. For example, Berger et al. (2020) tracked hedgehogs (*Erinaceus europaeus*) before, during and after a large music festival in a park, reporting substantial effects on movement, exceeding the impacts of existing habitat fragmentation. Similar effects have been documented following other social activities (see Kölzsch et al., 2023; Perona et al., 2019; Spelt et al., 2019), for example responses to New Year's Eve fireworks (Hoekstra et al., 2024). Dynamic disturbances also include human‐caused disasters (e.g. oil spills), armed conflicts (Gaynor et al., 2016; Russell et al., 2024) and moving infrastructures such as wind or tidal turbines which can be switched on and off, or floating offshore wind turbines. COVID‐19 lockdowns resulted in a period of drastically reduced human mobility (an ‘anthropause’), creating unusual environment conditions for some animals that had adapted to normal human mobility rhythms (Rutz et al., 2020; Tucker et al., 2023); see also the related example of the Chernobyl Exclusion Zone (Dombrovski et al., 2022). Dynamic disturbances also include the transitionary period from natural habitats to human‐modified environments. Construction activities have been shown to impact animal movement routes (Leblond et al., 2013). In particular, Lesmerises et al. (2013) and Skarin et al. (2015) showed that active road construction work has greater effects on movement than just the presence of the road itself for wolves (*Canis lupus*) and reindeer (*Rangifer tarandus*), respectively. The noise and vibrations emitted during the construction process of offshore wind farms can also cause significant disruption to marine animals, including behavioural changes and disturbance (Brandt et al., 2011; Herbert‐Read et al., 2017; Whyte et al., 2020), although after construction wind farm foundations may become attractive artificial reef systems (Werner et al., 2024).

Static and dynamic changes often interact, and this interplay is a key determinant of the degree of disturbance introduced to the environment (Figure 1). Roads, for example, are static features that alter the physical environment through soil compaction, increased surface temperature, and changes in surface‐water flow (Trombulak & Frissell, 2000), and can act as major barriers to animal movement. This modification is further intensified by the dynamic elements, such as humans and vehicles, that use the road, causing disturbance and animal‐vehicle collisions (see also Rutz, 2022b). Studying the impacts of human‐modified environments on wildlife thus requires examination of both static and dynamic components, and their interacting effects.

### 2.2 Features of human‐modified environments as drivers of animal movements

#### 2.2.1 Redistribution of resources

Human‐modified environments have led to drastic changes in resource distributions, limiting some natural resources while making novel resources available. Land‐cover change may expose animals to novel conditions and is among the leading causes of biodiversity loss (e.g. IPBES, 2019; Newbold et al., 2015; Wacher et al., 2023).

In human‐modified environments, resource distribution is shaped by novel resources, such as food waste or human infrastructure, which may lead to altered behavioural or movement choices by animals (Sih, 2013). Anthropogenic subsidies (e.g. landfill sites, fishery discards or domestic waste bins) tend to be predictable in space and time, leading to animals foraging in and around concentrated areas (Oro et al., 2013; e.g. spotted hyenas (*Crocuta crocuta*), Yirga et al., 2015; water monitor lizards (*Varanus salvator*), Uyeda et al., 2015; black‐backed gulls (*Larus fuscus*), Spelt et al., 2019). Seabirds are known to show an impressive ability to adapt their diets to human‐induced changes (Bicknell et al., 2013; Griffin et al., 2017; Guerra et al., 2022). Similarly, human infrastructure (e.g. roads, buildings, dams and street lamps) may also be perceived as a resource that functionally resembles natural physical structures, sometimes leading to unreliable cues for animals when determining habitat quality (Imlay et al., 2019; Nisi et al., 2022; Plummer et al., 2016; Sih, 2013). These predictable resources can also impact seasonal movements, as illustrated by generations of white storks (*Ciconia ciconia*) changing their migratory behaviour to optimally use human‐made resources (Gilbert et al., 2016). Spatial reconfiguration of habitat patches can also alter animal distributions, even if the composition of the landscape itself does not change (Macdonald & Johnson, 2015).

#### 2.2.2 Altered climate conditions and thermal landscapes

In addition to direct changes to the landscape, human activities are known to impact climate (Calvin et al., 2023). Consequently, new climatic and weather conditions can strongly shape species distributions, which is further compounded by changes to land cover and resource distributions. Maintaining body temperatures close to physiological optima is key for animal homeostasis and, to different degrees, all animals rely on their surrounding environment (‘microclimate’, see review in Kemppinen et al., 2024) for thermoregulation. Thus, under global warming, distributions of many species are expected to shift (Poloczanska et al., 2013; Sunday et al., 2012, but see also: Fuchs et al., 2024). For example, ocean warming is predicted to cause Arctic whales to move northward (Chambault et al., 2022) and reduce grey reef shark (*Carcharhinus amblyrhynchos*) residency to coral reefs (Williamson et al., 2024). Maximum dive depths of blue sharks (*Prionace glauca*) have been found to decrease with high sea temperatures and decreasing dissolved oxygen (Vedor et al., 2021). Many terrestrial mammals from arid environments are expected to expand their home ranges if precipitation decreases with climate change (Bennitt et al., 2018). Besides global shifts in temperatures, extreme weather and climatic events are increasing in frequency, including heat waves or strong storms associated with high‐speed winds (Newman & Noy, 2023). These events have disruptive effects on abiotic and biotic ecosystem elements, with either immediate or long‐term consequences. The abrupt and disruptive nature of extreme weather can strongly modify the distribution of resources (e.g. Amoroso et al., 2020; Soriano‐Redondo et al., 2016) and trigger avoidance or attraction behaviours. This has been documented in white‐tailed deer (*Odocoileus virginianus*) avoiding hurricanes (Abernathy et al., 2019), predators waiting for and catching fleeing prey (Nimmo et al., 2019) or seabirds flying into the eye of storms to reduce risk (Lempidakis et al., 2022). Where habitats are thermally heterogeneous, which is common for most terrestrial species, animals may choose between different available microclimatic conditions to buffer climate change impacts (Beever et al., 2017). Such behavioural thermoregulation can be observed for example in giant anteaters (*Myrmecophaga tridactyla*), where temperature‐dependent adjustments in activity patterns and habitat preferences are evident (Giroux et al., 2023).

#### 2.2.3 Amplified risk landscape

Humans can be viewed as super‐predators that can strongly disturb animal movements and behaviour, for example top predators may avoid areas with humans (Serra‐Medeiros et al., 2021; Suraci et al., 2019) and human voices may generate stronger fear responses than lion sounds across several taxa in the South African savanna (Zanette et al., 2023). Animals therefore have to face an activity‐risk trade‐off, similar to how prey species have to navigate the landscape of fear created by their predators. Examples include sounds that people generate during outdoor recreation activities which cause strong anti‐predator responses (Zeller et al., 2024). Both real (e.g. hunting) and perceived (e.g. hiking) risks cause behavioural changes, leading to similar costs and potential maladaptation in wildlife populations (Courbin et al., 2022). More intense human activities or extreme anthropogenic disturbances such as armed conflicts (Russell et al., 2024), fireworks (Hoekstra et al., 2024) or other sporadic bursts of anthropogenic noise (Hastie et al., 2021) can impact movements and mortality risks, especially in cases of prolonged exposure. Conversely, drastic reductions in the number and distribution of humans, such as during the COVID anthropause (Rutz et al., 2020), have led to changes in the movement behaviour of various wildlife species worldwide, albeit with a high degree of context dependence (Bates et al., 2021; Tucker et al., 2023; see also Burton et al., 2024).

#### 2.2.4 Effects on movement capacity and the energy landscape

Human‐made structures, such as roads, fences, wind turbines, oil platforms, dams, buildings and bridges, can impact the capacity of animals to move. Linear structures such as roads, railways or fences act as barriers and contribute to habitat fragmentation (Forman & Alexander, 1998; van der Ree et al., 2015). For example, Jones et al. (2022) indicate that both fences and roads are affecting pronghorn (*Antilocapra americana*) movements and resource use. Vertical structures such as wind turbines, skyscrapers or towers (Loss et al., 2014) create disturbances that have been responsible for displacing animals (Masden et al., 2009) and increasing mortality through collisions (Loss et al., 2013). Human‐made structures can also modify the energy landscape for movements by facilitating or preventing movements around structures. Human‐built structures can, for example, change the space‐use patterns of flying birds by altering the airflow around buildings and hence the profitability of the airscape (Shepard et al., 2016). Wolves (*Canis lupus*) have also been shown to preferentially move along linear tracks opened for oil and gas exploration (Dickie et al., 2017).

#### 2.2.5 Sensory pollution and navigational capacity

Movement is guided by sensory cues that allow individuals to navigate (Dusenbery, 1992). Both human activities and infrastructure can create sensory pollution affecting animal movement (Dominoni et al., 2020). Many examples illustrate how human‐induced changes can interfere with formerly adaptive and reliable natural cues guiding animal movement and habitat selection, which may no longer be associated with positive outcomes in novel human‐modified environments (Schlaepfer et al., 2002). These are known as maladaptive responses. Artificial lights can attract insects that many species feed on, alter predator–prey dynamics (e.g. cougar [*Puma concolor*] and mule deer [*Odocoileus hemionus*]; Ditmer et al., 2021) and cause navigational problems by mimicking natural stimuli that guide movement in turtle hatchlings (Tuxbury & Salmon, 2005) and fledgling seabirds. Similarly, artificial sound, such as from sonar and traffic, can interfere with movement and navigation in aquatic (Barcelo‐Serra et al., 2021) and terrestrial systems (Schaub et al., 2008), leading to sound pollution (te Velde et al., 2024). Beaked whales may react to naval sonar signals as they would to the sound of a predator, expending more energy in escape responses, risking stranding or decompression sickness (Simonis et al., 2020). Chemical pollution may also interfere with sensory perception and navigation in animals. The impacts of chemical pollution may be complex and multimodal, affecting multiple sensory domains used by animals for orientation (Halfwerk & Slabbekoorn, 2015). Plastic pollution, and its associated chemical pollutants, also shows complex and concerning impacts on wildlife, for example by altering the navigation capacity of animals (e.g. attracting marine turtles, Pfaller et al., 2020), or immobilising *Daphnia* species (Bucci et al., 2020). More generally, human activities and disturbances, and environmental conditions in human‐dominated areas such as urban areas, can be stressful for animals, and evidence is accumulating that they may directly and/or indirectly affect cognitive performance (see Chow et al., 2024 and references therein).

#### 2.2.6 Novel species interactions

Intra‐ and inter‐specific interactions fundamentally affect animal movements (Nathan et al., 2008), and their quantitative investigation promises significant advances in our understanding of spatial and community ecology (reviewed in Costa‐Pereira et al., 2022). The challenge in human‐modified areas is that human actions are profoundly affecting biotic interactions, through the addition of non‐indigenous species, the spread of invasive species (Rilov et al., 2024; Vilà et al., 2011), and the removal of native species (e.g. removal of large predators). Furthermore, key conservation actions such as rewilding are increasingly using functionally equivalent but non‐native species (Svenning, Buitenwerf, et al., 2024). Thus, predicting animal movements in human‐modified environments will require the inclusion of the effects of potentially new or non‐native biotic interactions. In addition, a recent review by Gaynor et al. (2024) highlights that understanding the spatial‐social mechanisms linking human disturbances to population outcomes is key to mitigating undesired consequences of human‐related changes. The latter can trigger a cascade of process alterations that can impact numerous species and their inter‐species interactions. Examples include the increased predation of migratory fish at river barriers (Mensinger et al., 2024), or human infrastructure attracting predators (e.g. common ravens *Corvus corax*) to previously marginal habitats, resulting in hyper‐predation of sensitive prey species such as greater sage‐grouse (*Centrocercus urophasianus*; Harju et al., 2021) and desert tortoises (*Gopherus agassizii*; Kristan & Boarman, 2007).

### 2.3 Impacts of human‐modified environments

Tucker et al. (2018) showed that some aspects of the movement behaviour of terrestrial mammals are strongly associated with the human footprint index—a measurement of human pressure on landscapes, based on a combination of multiple anthropogenic variables, including human population density and infrastructure (Venter et al., 2016). Specifically, there was a general tendency towards reduced movements in human‐dominated areas, albeit with very large variability within and between species. In this section, we briefly review the current knowledge (or lack thereof) on the impacts of these changes in movements in response to human influence.

#### 2.3.1 Anthropocene winners and losers

The resilience and adaptation exhibited in the face of changing conditions in human‐dominated environments diverge remarkably across species, yielding a wide variety of behavioural responses and fitness outcomes. For example, in response to human changes, animals may modify their movement behaviours, which can ultimately have positive, negative or neutral influences on individual fitness (Matthiopoulos, 2022). Understanding what makes a species a winner or a loser in the Anthropocene is currently a considerable and unsolved challenge. In general, individuals can be considered *Anthropocene winners* when responses caused by human disturbances yield improved survival and/or reproductive rates. Species with prior experience of similar cues or conditions (typically generalist species with a wide behavioural repertoire) tend to perform better in human‐modified environments (Sarkar & Bhadra, 2022). This phenomenon is evident among numerous examples of generalists, such as coyotes, foxes, bears, leopards, omnivorous tetra fishes and gulls that are shifting into human‐modified environments and adapting to human disturbances and infrastructure (Hody & Kays, 2018; Spelt et al., 2019). These species can actively colonise human‐modified habitats because of the availability of valuable resources (such as food or nest sites) or protection from threats such as predators. Predictable, high‐calorie food sources can increase reproduction and therefore fitness of these species (Gutmann Roberts et al., 2019; Newsome et al., 2010; Strum, 2010)—though this often comes at the expense of other species, increasing competition and reducing community diversity (McKinney, 2006; Oro et al., 2013; Shochat et al., 2006). This process can alter community composition, as seen in agricultural areas and urban centres, often with biodiversity homogenisation dominated by ‘human‐adaptable’ species (Clavel et al., 2011; Ducatez et al., 2018). Conversely, *Anthropocene losers* experience declines in survival and reproduction due to responses caused by human modifications and activities. Among them, some species change their movements, by avoiding areas dominated and disturbed by human activity or infrastructure, leading, for example, to reduced home ranges (e.g. Perona et al., 2019) or changes in migration (Gilbert et al., 2016). This may negatively affect species fitness at different spatial and temporal scales, for example, as a consequence of increased movement costs. Similarly, species switching their distributions to human‐modified areas can also experience negative consequences such as increased stress (Chow et al., 2024; Rolland et al., 2012), elevated mortality (e.g. due to collision, higher disease incidence) and impaired reproductive success (Romano et al., 2006). This can be understood through the concept of ecological traps, where animals mistakenly prefer human‐modified environments where their overall fitness is lower because of unreliable cues (Hale & Swearer, 2016). Linking ecological trap occurrence to population demography is a key step in improving our understanding of this phenomenon.

#### 2.3.2 Delayed and cumulative effects

Human impacts on individuals and species may be spatially distant or temporally lagged from their causes. For example, for marine animals washed up on beaches, the location, time or cause of mortality may be distant in space and/or time. Similarly, migratory animals experience a wide range of habitats and disturbance regimes as they move over large distances and may display the consequences of such exposure at a different place and time, possibly having accumulated sub‐lethal disturbances until the effects are manifested (Russell et al., 2024). These displaced effects warrant consideration because they may imply that the spatiotemporal zone of human impact is much greater than we assume (Niebuhr et al., 2022). Impacts of co‐occurring disturbance sources can accumulate along different dimensions (e.g. multiple types of disturbance, multiple features of the same type, trophic accumulation and time accumulation) and lead to different synergies (e.g. additive, multiplicative and mitigative). Hence, studies have shown that understanding the cumulative impacts of human disturbance in space and time is key to mitigation (Ellis‐Soto et al., 2023; Johnson & St‐Laurent, 2011; Niebuhr et al., 2022; Oliver et al., 2024).

## 3 MODELLING AND PREDICTING ANIMAL MOVEMENTS IN HUMAN‐MODIFIED ENVIRONMENT—CHALLENGES AND POTENTIAL SOLUTIONS

Predicting future changes in animal movements and distribution is crucial for providing conservation solutions. Robust predictions allow any potential negative impacts of different land‐use and conservation management scenarios on movements and distributions to be anticipated and mitigated against. However, predicting animal movement is notoriously difficult, even under static environmental conditions, let alone in rapidly changing human‐influenced environments and novel ecosystems. We urgently need practical methodological frameworks to measure, model, predict and evaluate animal movements and distribution under human‐modified environments (Fieberg et al., 2024). Here, we discuss how best to account for novel conditions in model predictions and address what is missing from current methodological tools.

In the presence of rapid environmental change, our models must make predictions outside the range of historically observed scenarios. Predictive models must consider that future environmental and ecological contexts may be different from those in which the data were collected. To achieve this, we need a good understanding of animal decision‐making, especially when animals operate across spatiotemporal scales, in response to multiple life‐history priorities, balancing demands for survival, reproduction and dispersal. Unlike inanimate objects, whose movement can be described by physical laws and mathematical models to a great degree of precision, animal movements are complex, varied, often hard to detect, and fundamentally characterised by agency and individual variation (Hawkes, 2009; Hertel et al., 2020; Shaw, 2020), posing a significant challenge to building predictive models (Chatterjee et al., 2024; Muff et al., 2020). This context dependence has to be carefully examined when we aim to construct generalisable models of movement and distribution (Matthiopoulos et al., 2011). Ultimately, predicting movement in human‐modified environments requires mechanistic models that use functional relationships derived from first principles of movement and also utilise information from historically contrasting environmental scenarios in different geographical regions and time periods.

Animal movement and distribution models are often based on approaches that correlate animal locations with different variables (e.g. fixed or changing environmental features or locations of other animals), potentially using the resulting models to predict similar correlations at a future time or in another system. Correlative examples include resource selection functions (RSFs, Manly et al., 2002) and species distribution models (SDMs) fitted by maximum entropy (Elith & Leathwick, 2009) or by likelihood (Manly et al., 2002). When using correlative approaches to make predictions, the assumption is that those correlations will be the same in the future or in different environmental contexts. Importantly, this assumption also underlies sophisticated predictive models built using machine learning and AI. However, this assumption rarely holds (Matthiopoulos et al., 2011; Yates et al., 2018), with organisms in different environmental contexts behaving in fundamentally different ways from what traditional correlative models predict. It may be useful to expand here on the difference between geographic and environmental space when fitting SDMs or RSFs (Matthiopoulos, 2022). If the novel (anthropogenic) environment contains a new combination of environmental covariates, or values beyond those used to fit the model, then these missing predictors can lead to biased estimators of causal effects and poor out‐of‐sample predictions (Rinella et al., 2020). However, if the new environment contains combinations of covariates within the bounds of the model fitting (even if they are in a new geographic area), then the predictions should be more robust. Importantly, the key aim is not to describe the existing data as well as possible, but rather to understand the underlying mechanisms and have models that can predict under novel situations.

A possible way to remedy this is to build mechanistic models of the movement decisions made by animals, ideally based on first principles of movement and functional relationships, and then project them forwards to predict space‐use patterns on a broader spatiotemporal scale (e.g. Signer et al., 2024). However, this can cause a different problem, whereby models describing movement decisions on one time scale can become inaccurate if scaled up to a broader time scale (Potts & Börger, 2023). For example, models detailing the correlates of movement between successive 2‐hourly location fixes may wildly mis‐predict space use over an entire month (Potts et al., 2022). It is tempting for researchers only to assess predictions at the spatiotemporal scale on which models are fitted: In our example of 2‐hourly fixes, one could simply assess the model by looking at how well it predicts the next location fix (e.g. using methods outlined by Auger‐Méthé et al., 2021; Fieberg et al., 2018). This might lead to ostensibly better results, but it hides the fact that any accurate description of movement ought to remain accurate when scaled up. The new line‐up method presented by Fieberg et al. (2024) is an example of how predictions over longer time scales can be evaluated. Moreover, ecosystems are dynamic, comprising many interacting and fluctuating animal populations. Dynamical systems often include feedback effects, making regression models (e.g. generalised linear models and their variants looking at the response of one species to another) insufficient (Riotte‐Lambert & Matthiopoulos, 2020). Rather, each component affects, and is affected by, the others in a dynamic network of interactions. As developed for intra‐specific interactions (Milner et al., 2021; Niu et al., 2016; Potts & Schlägel, 2020; Schlägel et al., 2019), we must increasingly capture multi‐way species interactions within predictive models.

Discrepancies between predictions and observations can reveal the biological features missing from the models (Potts et al., 2022). The idea of building a model describing movement on one spatiotemporal scale, and then using it to predict spatial patterns on a broader scale, is called a process‐based approach (Avgar et al., 2016; Malishev & Kramer‐Schadt, 2021) or sometimes a mechanistic approach (Potts & Lewis, 2014). By increasing the mechanistic content of statistical models, we can move towards a situation where we can extrapolate the movement of animals to multiple scales and contexts. Progress on statistical techniques such as step‐selection functions (SSF) (e.g. Klappstein et al., 2022, 2024; Potts & Börger, 2023) and state‐space models (e.g. Newman et al., 2023; Patterson et al., 2008) allows these tools to be increasingly process‐based. By modelling the decisions animals make whilst they move, process‐based models can implicitly incorporate the timescale over which space‐use patterns vary. If the underlying environment is changing faster than the space‐use patterns emerge, then a process‐based model could capture this perpetually transient animal space use. To account for movement decisions at different temporal scales, another approach attempts to model movement and utilisation distributions jointly (Michelot, Blackwell, et al., 2019). This method uses stochastic models derived from Markov Chain Monte Carlo methods in discrete time (Michelot et al., 2020; Michelot, Blackwell, et al., 2019) and continuous time (Michelot, Gloaguen, et al., 2019), allowing joint inference at multiple scales (Blackwell & Matthiopoulos, 2024).

**FIGURE 2:**
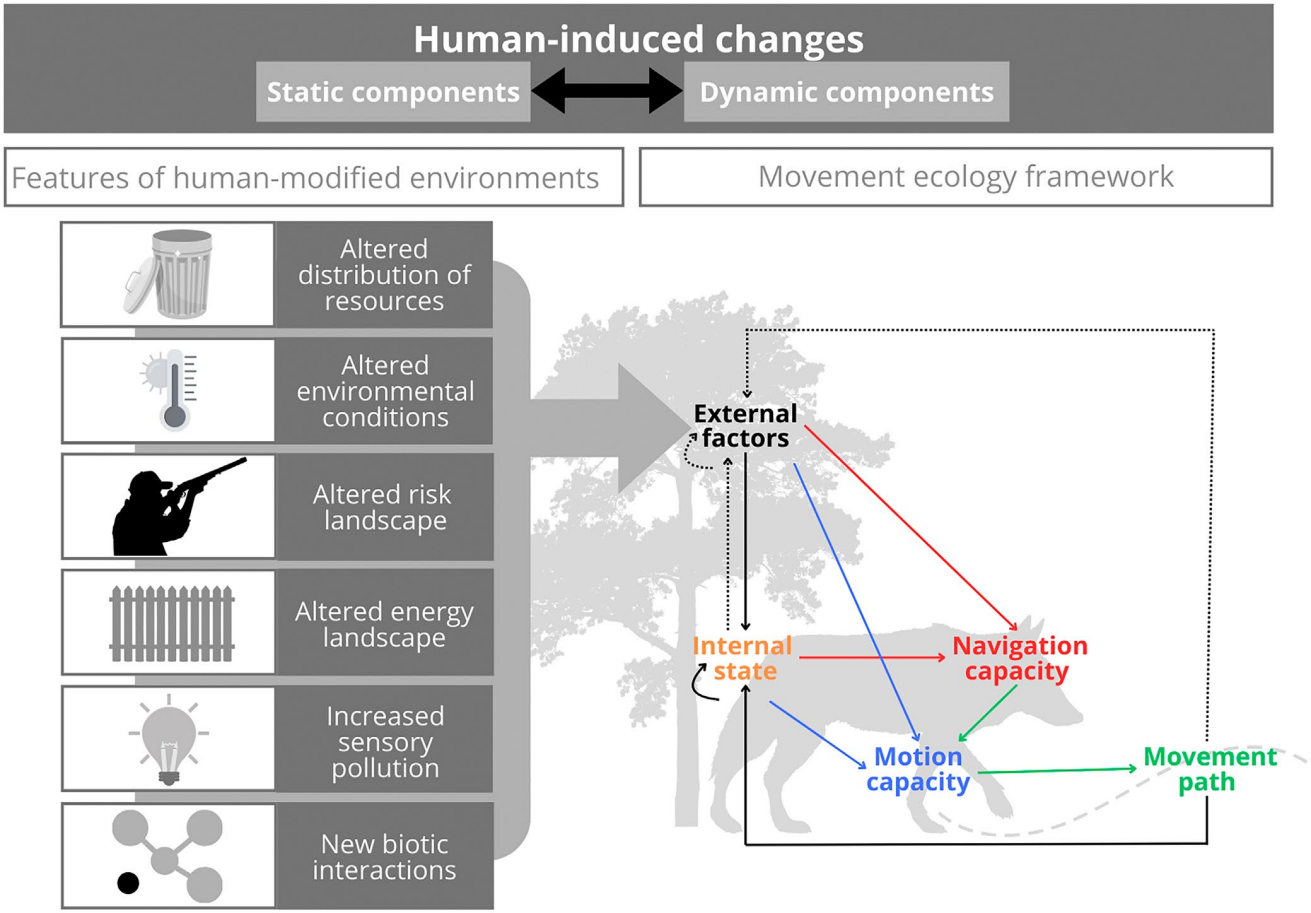
Human‐induced changes have static and dynamic components that interact to create human‐modified environments with features that may alter animal movements. Here, we represent the main features of human‐modified environments upon the movement ecology framework (Nathan et al., 2008). A detailed description of each feature and more examples of its effects on animal movement are provided in the text (Section 2.1).

It is important to mention that, within process‐based models, the ‘process’ itself is generally fitted to empirical data using correlative methods (Potts & Börger, 2023). For example, mechanistic (aka process‐based) models of movement decisions are often fitted by correlating movement with environmental features, for example using hidden Markov models (McClintock & Michelot, 2018) or step‐selection analyses (Avgar et al., 2016; Potts & Lewis, 2014). A detailed consideration of the movement capacity of different species in different energy landscapes (Shepard et al., 2013) can markedly increase our predictive ability of where and when animals will move, as for example, in soaring birds under changing meteorological conditions (Shepard & Lambertucci, 2013). Thus, explicitly adding into the models how human‐modified environments affect the drivers of animal movements (see Figure 2) will be fundamentally important. Likewise, there is increasing realisation that human mobility (see Section 2.1) affects wildlife movements in ways that are not fully captured by land‐cover data or compound indices alone (Ellis‐Soto et al., 2023; Oliver et al., 2024). Such correlations (i.e. the features that drive movement decisions) are emergent features of some underlying process, such as physiological limitations or life‐history needs. Thus, if we want to use mechanistic models to make better predictions for changing environments, we need sufficiently detailed descriptions of animal movement to capture space use with accuracy, which is often challenging but sometimes achievable (Merkle et al., 2017; Moorcroft et al., 2006).

To make this more generally possible requires drawing on the widest possible historical information gathered over the last 50+ years of wildlife tracking, providing a large range of data across varied environmental contexts and human‐impact scenarios, in different geographical regions and at different times. We have to make the most of empirical data by extracting maximum information from voluminous and multi‐type data collected across different ecological contexts. Furthermore, we must better use experimental approaches to test mechanistic hypotheses and corroborate links between theoretical predictions and empirical observations (Lustenhouwer et al., 2023; Ranc et al., 2021). Finally, to remain useful in understanding animal movement in human‐modified environments, mechanistic models must be no more complicated than the available data can support. Yet, they also need to be expandable enough to admit more features as data accumulate and thus allow us to fit our models to a multiplicity of environmental contexts and diverse data sets, including positional (from GPS record, e.g. Berger et al., 2020 or citizen science, e.g. Rueda‐Uribe et al., 2024), behavioural (e.g. Chakravarty et al., 2019) or energetic (e.g. Klappstein et al., 2022; Shepard et al., 2013).

Overall, models aiming to predict animal movements in changing, human‐modified environments need to move beyond purely correlative approaches towards a dynamical systems perspective, which is much closer to the reality of ecosystems. To achieve this, we need to: (1) increase the mechanistic content of our models based on empirical movement data, (2) fit our models to a large range of integrated data sets, from historically contrasting environmental scenarios, and (3) better integrate theory and empirical observations through experimental approaches.

## 4 GETTING PRACTICAL—TURNING MOVEMENT MODELS AND PREDICTIONS INTO POLICY AND MANAGEMENT STRATEGIES

Predictions of where, when, how and why animals move can improve policy and management decisions (Allen & Singh, 2016; Hays et al., 2019; Yanco et al., 2025). For instance, using movement data to design wildlife corridors in the Yellowstone to Yukon region has significantly improved connectivity for migratory species, illustrating how robust predictions can translate into valuable conservation outcomes (Hebblewhite & Merrill, 2009). That said, the full potential of incorporating movement predictions into conservation actions is often not achieved (Fraser et al., 2018; Katzner & Arlettaz, 2020). To overcome this translation gap, studies have highlighted the necessity of movement scientists and practitioners to co‐design projects and fix common aims and priorities (Kadykalo et al., 2021; Nuijten et al., 2023). In this section, we showcase opportunities to improve the practical application of movement predictions within novel human‐modified environments. First, we highlight the importance of identifying the specific features of human disturbances (and hence also management actions) and the specific mechanisms through which they affect animal movements (e.g. by modifying the sensory or movement capacity of individuals; see Section 2, Figures 1 and 2). Second, establishing these causal links and identifying the mechanisms is crucial, as developing effective predictive movement models requires using mechanistic dynamical system models instead of correlative approaches (see Section 3). Adopting this framework right at the start of the project, in joined meetings between modellers and practitioners, will allow co‐development of specific management‐relevant predictive movement models and turning model predictions into effective management strategies. Furthermore, we discuss here also three further challenges and opportunities to address to bridge the research to management implementation gap: the need to carefully consider the spatiotemporal scales of predictions and conservation decisions; the importance of understanding the presence of significant data gaps that can lead to erroneous inferences; and the use of management actions as opportunities to collect highly relevant data for building predictive models and/or for testing model predictions experimentally.

### 4.1 Considering spatiotemporal scales in conservation decisions

Consideration of spatiotemporal scale is crucial when using movement models for conservation decisions. Indeed, positive effects of mitigating management decisions may only have an impact if implemented on a particular spatiotemporal scale because the effectiveness of management actions depends on their implementation at appropriate, biologically meaningful scales. For example, particular challenges may arise when animal movement extends beyond a spatially delimited management unit with planning and decision responsibilities (Bénard et al., 2024; Meisingset et al., 2018). Such mismatches may lead to mismanagement with detrimental effects on ecosystems (Delsink et al., 2013).

Second, model predictions not only need to fit well with observations, but must also connect well to the local context of management sites (Fortin et al., 2020) and be at the appropriate, management‐relevant scale. For example, model predictions at daily or weekly temporal scales will likely be ineffective for management programmes operating at yearly or even multi‐decadal time scales, as is the case for many rewilding projects. Likewise, significant changes to a population or ecosystem may only be detectable after a considerable time lag.

Failure to achieve this linkage risks creating misunderstandings, inefficiencies, and distrust between modellers and practitioners and can risk scientific recommendations not being fully incorporated into decision‐making (Delsink et al., 2013; Selier et al., 2015). Whereas predictive movement models can provide key information for management strategies, understanding and choosing relevant spatiotemporal scales to obtain effective model outcomes is often challenging in conservation contexts (Delsink et al., 2013). Thus, while we keep improving our models through increased process‐based predictions (Potts et al., 2022), all these model development aspects must be communicated clearly to decision‐makers and supported with appropriate evidence to facilitate successful conservation decisions (see also Nuijten et al., 2023). Communication is also crucial as an important further challenge lies in the temporal mismatch between the time required for the development of good predictive movement models and the urgency of implementing management actions which managers and practitioners often face. To effectively overcome these issues of scales, it is hence crucial that ecological modellers and managers co‐develop from the start the model aims and modelling pipeline in parallel with the management/conservation aims, including timelines and procedures to jointly assess and refine model predictions and the effectiveness of management actions (e.g. following an adaptive management framework—see Section 4.3).

### 4.2 Data deficiencies

Whilst there has been an increase in our ability to collect large movement and other bio‐logging data sets for an ever‐expanding range of species (Holton et al., 2021; Kays et al., 2015; Nathan et al., 2022; Williams et al., 2020), when converting movement predictions to conservation actions, we must acknowledge that our current understanding of animal movements is highly incomplete (e.g. see fig. 1 in Tucker et al., 2018). In particular, the movements of some taxa are rarely studied (amphibians, reptiles and invertebrates) compared with others (birds and mammals). There are also temporal data deficiencies due to technological constraints (e.g. battery life) and practical challenges in attaching and retrieving tracking devices (Crane et al., 2021), often related to ethical considerations with tags being too big for species (e.g. Symons & Diamond, 2019). There are also many data‐deficient habitats and regions, with a high concentration of movement ecology studies based in the Global North. To maximise the reliability of movement predictions, it is important to improve our data collection in the field, especially for the non‐studied species. Novel technologies can help overcome these needs thanks to the miniaturisation of technologies for studying smaller species and the use of video/AI technology for movement of species too small to fit trackers (i.e. insects; van Klink et al., 2022; Ratnayake et al., 2022). Nevertheless, collecting such data involves large costs and the benefits of obtaining more data must be carefully evaluated (McGowan et al., 2017). Sampling bias should always be considered in bio‐logging studies because tagged animals may not be fully representative of the wider populations for which we hope to draw inferences, as detailed in the STRANGE framework (‘Social background; Trappability and self‐selection; Rearing history; Acclimation and habituation; Natural changes in responsiveness; Genetic make‐up; and Experience’: Webster & Rutz, 2020; see Marshall et al., 2020 for an example on king cobra *Ophiophagus hannah*). Moreover, despite recent advances in data‐sharing through the development of dedicated repositories for movement data, such as the Movebank repository (Kays et al., 2021), many movement datasets remain effectively hidden from further use (Crane et al., 2021; Davidson et al., 2025; Rutz, 2022a). Like animal movement data, key covariates are also usually missing to develop good predictive models; this concerns especially fine‐scale human‐related dynamic covariates (e.g. road or trail traffic, land‐use change across years, mines and shipping activity). Recent efforts to understand the effects of human mobility on wildlife, for example, through analysing the impact of COVID‐19 lockdown measures (Rutz et al., 2020) on animal movements, have highlighted the crucial need for high‐resolution human mobility data (Ellis‐Soto et al., 2023; Oliver et al., 2024).

### 4.3 Management and conservation actions as opportunities for new data and experimental tests of model predictions

Many conservation and management interventions markedly affect the distribution of resources (e.g. supplemental feeding and habitat conversion) or the conditions experienced by animals (e.g. translocations), hence offering quasi‐experimental conditions to collect data that would otherwise be very difficult or expensive to collect. For example, Silovský et al. (2024) used translocations of GPS‐tagged red deer (*Cervus elaphus*) to understand their homing behaviour and orientation. Similarly, Ranc et al. (2021) used supplementary winter feeding as experimental tests for memory‐based foraging decisions. Translocations and experimental feeding often occur in highly human‐modified environments and should be better used to design research and management actions. Such studies provide precisely the sort of data required for building mechanistic models (see Section 3) or for testing model predictions. Considerable effort is currently also directed towards restoration and trophic rewilding actions (Burak et al., 2024; Maes et al., 2024), involving extensive habitat modifications as well as the creation of new species interactions (Svenning, Buitenwerf, et al., 2024), offering opportunities to collect key data to observe and predict animal movements in novel environments, as well as to test model predictions.

Another exciting opportunity is offered by current approaches used to manage ecosystems under climate change, namely RAD (*resist* the climate transformation; *accept* the transformation and manage the current state; *direct* the system towards a novel state; Williams & Brown, 2024) and adaptive management frameworks in general (Månsson et al., 2023). These approaches are stepwise iterative processes which focus particularly on mechanisms and predictions, with prediction, monitoring and assessment of predictions being the basis for management. Hence, we suggest that these offer an excellent framework for integrating predictions of animal movements in novel environments. They offer a coherent management approach whereby management interventions are planned, their effects predicted into the future, the effects are monitored and evaluated, and the subsequent intervention is adapted based on the knowledge gained from assessing the predictive ability of the previous intervention. Such an iterative approach mirrors very well the iterative approach proposed by recent research to develop improved predictive movement models (Potts & Börger, 2023). Nevertheless, current research has highlighted that adaptive management approaches face critical challenges in rapidly changing environments, such as those caused by land‐use and climate change (Månsson et al., 2023). Hence, the inclusion of mechanistic predictive movement models, derived from first principles of animal movement, into adaptive management approaches promises to be of mutual benefit to movement ecology and conservation.

## 5 CONCLUSIONS

Understanding and predicting animal movements is of crucial importance for our comprehension of ecological and evolutionary processes and provides key evidence for conserving and managing species and ecosystems, especially in the current era of rapid human‐caused environmental change. There has been tremendous progress in our ability to collect large animal movement and bio‐logging datasets at rapidly increasing resolution and in modelling large and complex datasets, thanks to progress in technology and mathematical modelling, similar to wider trends in ecological research (McCrea et al., 2023). Notwithstanding this progress, predicting animal movements and distributions, especially in rapidly changing, human‐modified or novel environments, remains challenging and is an active area of research.

To progress, in this review we first discussed the defining features of human‐modified environments and the resulting consequences on the drivers of movement. We then posited that, to be able to incorporate robust predictions into management and policy, models aiming to predict animal movements in human‐modified landscapes need to be able to make predictions for novel contexts, outside the range of existing data.

First, we argued for a thoughtful and focused approach to data collection. We need to integrate different types of data at appropriate scales, creating nested hierarchies of data. Careful and targeted data collection must also acknowledge biases in observations, with implications regarding the indiscriminate use of citizen science data, for example. Such data offer extraordinary potential for ecological research and are rapidly increasing in availability and use (Dennhardt et al., 2015; Fuentes et al., 2023; Rueda‐Uribe et al., 2024; Yun et al., 2024), but their use in developing predictive movement models will require careful scientific supervision and calibration. We must also put more effort into increasing the number of different species studied and increasing the number of studies in human‐dominated or novel environments—such data are critically lacking. Furthermore, current research systems tend to favour isolated, short‐term studies usually focused on a single study system. Instead, we need funding for coordinated multi‐system projects with multiple principal investigators (including modellers and statisticians alongside field experts on each study system) recording data of multiple types (see also the call for data integration by McCrea et al., 2023).

Second, we argued for a careful and meticulous incorporation of biological mechanisms in predictive movement and distribution models, by using functional relationships derived from first principles of movement. This requirement leads to a second cautionary note, against the indiscriminate use of machine learning for the analysis and predictive modelling of movement data. Notwithstanding the enormous potential and advantages of machine learning, also for movement studies (e.g. Rieber et al., 2024; Schoen et al., 2025; Sueur, 2023; Wijeyakulasuriya et al., 2020), we have to be able to supervise the models that are constructed to generate robust predictions (see also McCrea et al., 2023; on the importance of having a methodological driver underpinning the use of machine learning and AI models, and Tuia et al. (2022) on the importance to integrate ecological knowledge into machine learning models). Our key aim moving forward should not be to describe the existing data as well as possible, but rather to understand the underlying mechanisms driving movement patterns. This is crucial in developing models with robust predictive ability for novel future change scenarios.

In conclusion, we provide a framework to better understand and predict animal movements and distributions in dynamic and often novel environments. Robust predictions are crucial to produce reliable management and policy‐relevant evidence and predictions in the Anthropocene. The recommendations presented here, coupled with the impressive technological and methodological developments in the field, highlight the exciting opportunities now available to advance the field of movement ecology into a more predictive science.

## AUTHOR CONTRIBUTIONS

All authors contributed to the development and writing of this manuscript. The manuscript was conceived by Luca Börger in discussion with all authors at the BESMove 2022 meeting at the University of Glasgow on ‘Understanding animal movement in human‐altered landscapes’, organised by the Movement Ecology Special Interest Group of the British Ecological Society. Our study brings together authors from a number of different countries, and different career stages. All authors contributed to the writing of the manuscript, led by Sara Gomez, Holly M. English and Luca Börger, aided by Jason Matthiopoulos and Jonathan R. Potts.

## CONFLICT OF INTEREST STATEMENT

The authors declare no conflict of interests.

## Data Availability

Data were not collected or analysed for the purposes of this review.

## References

[jane70040-bib-0001] Abernathy, H. N. , Crawford, D. A. , Garrison, E. P. , Chandler, R. B. , Conner, M. L. , Miller, K. V. , & Cherry, M. J. (2019). Deer movement and resource selection during hurricane Irma: Implications for extreme climatic events and wildlife. Proceedings of the Royal Society B: Biological Sciences, 286(1916), 20192230. 10.1098/rspb.2019.2230 PMC6939277 31771480

[jane70040-bib-0002] Allen, A. M. , & Singh, N. J. (2016). Linking movement ecology with wildlife management and conservation. Frontiers in Ecology and Evolution, 3, 155. 10.3389/fevo.2015.00155

[jane70040-bib-0003] Amoroso, C. R. , Kappeler, P. M. , Fichtel, C. , & Nunn, C. L. (2020). Water availability impacts habitat use by red‐fronted lemurs (*Eulemur rufifrons*): An experimental and observational study. International Journal of Primatology, 41(1), 61–80. 10.1007/s10764-020-00136-9

[jane70040-bib-0004] Arroyo‐Rodríguez, V. , Fahrig, L. , Tabarelli, M. , Watling, J. I. , Tischendorf, L. , Benchimol, M. , Cazetta, E. , Faria, D. , Leal, I. R. , Melo, F. P. L. , Morante‐Filho, J. C. , Santos, B. A. , Arasa‐Gisbert, R. , Arce‐Peña, N. , Cervantes‐López, M. J. , Cudney‐Valenzuela, S. , Galán‐Acedo, C. , San‐José, M. , Vieira, I. C. G. , … Tscharntke, T. (2020). Designing optimal human‐modified landscapes for forest biodiversity conservation. Ecology Letters, 23(9), 1404–1420. 10.1111/ele.13535 32537896

[jane70040-bib-0005] Auger‐Méthé, M. , Newman, K. , Cole, D. , Empacher, F. , Gryba, R. , King, A. A. , Leos‐Barajas, V. , Mills Flemming, J. , Nielsen, A. , Petris, G. , & Thomas, L. (2021). A guide to state–space modeling of ecological time series. Ecological Monographs, 91(4), e01470. 10.1002/ecm.1470

[jane70040-bib-0006] Avgar, T. , Potts, J. R. , Lewis, M. A. , & Boyce, M. S. (2016). Integrated step selection analysis: Bridging the gap between resource selection and animal movement. Methods in Ecology and Evolution, 7(5), 619–630. 10.1111/2041-210X.12528

[jane70040-bib-0007] Barcelo‐Serra, M. , Cabanellas, S. , Palmer, M. , Bolgan, M. , & Alós, J. (2021). A state‐space model to derive motorboat noise effects on fish movement from acoustic tracking data. Scientific Reports, 11(1), 4765. 10.1038/s41598-021-84261-2 33637805 PMC7910575

[jane70040-bib-0008] Bates, A. E. , Primack, R. B. , Biggar, B. S. , Bird, T. J. , Clinton, M. E. , Command, R. J. , Richards, C. , Shellard, M. , Geraldi, N. R. , Vergara, V. , Acevedo‐Charry, O. , Colón‐Piñeiro, Z. , Ocampo, D. , Ocampo‐Peñuela, N. , Sánchez‐Clavijo, L. M. , Adamescu, C. M. , Cheval, S. , Racoviceanu, T. , Adams, M. D. , … Duarte, C. M. (2021). Global COVID‐19 lockdown highlights humans as both threats and custodians of the environment. Biological Conservation, 263, 109175. 10.1016/j.biocon.2021.109175 34035536 PMC8135229

[jane70040-bib-0009] Beever, E. A. , Hall, L. E. , Varner, J. , Loosen, A. E. , Dunham, J. B. , Gahl, M. K. , Smith, F. A. , & Lawler, J. J. (2017). Behavioral flexibility as a mechanism for coping with climate change. Frontiers in Ecology and the Environment, 15(6), 299–308. 10.1002/fee.1502

[jane70040-bib-0010] Bénard, A. , Lengagne, T. , & Bonenfant, C. (2024). Integration of animal movement into wildlife‐vehicle collision models. Ecological Modelling, 492, 110690. 10.1016/j.ecolmodel.2024.110690

[jane70040-bib-0011] Bender, E. A. , Case, T. J. , & Gilpin, M. E. (1984). Perturbation experiments in community ecology: Theory and practice. Ecology, 65(1), 1–13. 10.2307/1939452

[jane70040-bib-0012] Benítez‐López, A. , Alkemade, R. , & Verweij, P. A. (2010). The impacts of roads and other infrastructure on mammal and bird populations: A meta‐analysis. Biological Conservation, 143(6), 1307–1316. 10.1016/j.biocon.2010.02.009

[jane70040-bib-0013] Bennitt, E. , Bonyongo, M. C. , & Harris, S. (2018). Cape buffalo (*Syncerus caffer caffer*) social dynamics in a flood‐pulsed environment. Behavioral Ecology, 29(1), 93–105. 10.1093/beheco/arx138

[jane70040-bib-0014] Berger, A. , Barthel, L. M. F. , Rast, W. , Hofer, H. , & Gras, P. (2020). Urban hedgehog Behavioural responses to temporary habitat disturbance versus permanent fragmentation. Animals, 10(11), 2109. 10.3390/ani10112109 33203020 PMC7697271

[jane70040-bib-0015] Bicknell, A. W. J. , Oro, D. , Camphuysen, K., C. J. , & Votier, S. C. (2013). Potential consequences of discard reform for seabird communities. Journal of Applied Ecology, 50(3), 649–658. 10.1111/1365-2664.12072

[jane70040-bib-0016] Blackwell, P. G. , & Matthiopoulos, J. (2024). Joint inference for telemetry and spatial survey data. Ecology, 105(12), e4457. 10.1002/ecy.4457 39475100 PMC11610709

[jane70040-bib-0017] Boughman, J. W. , Brand, J. A. , Brooks, R. C. , Bonduriansky, R. , & Wong, B. B. M. (2024). Sexual selection and speciation in the Anthropocene. Trends in Ecology & Evolution, 39(7), 654–665. 10.1016/j.tree.2024.02.005 38503640

[jane70040-bib-0018] Brandt, M. J. , Diederichs, A. , Betke, K. , & Nehls, G. (2011). Responses of harbour porpoises to pile driving at the horns rev II offshore wind farm in the Danish North Sea. Marine Ecology Progress Series, 421, 205–216. 10.3354/meps08888

[jane70040-bib-0019] Bucci, K. , Tulio, M. , & Rochman, C. M. (2020). What is known and unknown about the effects of plastic pollution: A meta‐analysis and systematic review. Ecological Applications, 30(2), e02044. 10.1002/eap.2044 31758826

[jane70040-bib-0020] Burak, M. K. , Ferraro, K. M. , Orrick, K. D. , Sommer, N. R. , Ellis‐Soto, D. , & Schmitz, O. J. (2024). Context matters when rewilding for climate change. People and Nature, 6(2), 507–518. 10.1002/pan3.10609

[jane70040-bib-0021] Burton, A. C. , Beirne, C. , Gaynor, K. M. , Sun, C. , Granados, A. , Allen, M. L. , Alston, J. M. , Alvarenga, G. C. , Calderón, F. S. Á. , Amir, Z. , Anhalt‐Depies, C. , Appel, C. , Arroyo‐Arce, S. , Balme, G. , Bar‐Massada, A. , Barcelos, D. , Barr, E. , Barthelmess, E. L. , Baruzzi, C. , … Kays, R. (2024). Mammal responses to global changes in human activity vary by trophic group and landscape. Nature Ecology & Evolution, 8(5), 924–935. 10.1038/s41559-024-02363-2 38499871 PMC11090811

[jane70040-bib-0209] Calvin, K. , Dasgupta, D. , Krinner, G. , Mukherji, A. , Thorne, P. W. , Trisos, C. , Romero, J. , Aldunce, P. , Barrett, K. , Blanco, G. , Cheung, W. W. L. , Connors, S. , Denton, F. , Diongue‐Niang, A. , Dodman, D. , Garschagen, M. , Geden, O. , Hayward, B. , Jones, C. , … Ha, M. (2023). IPCC, 2023: Climate Change 2023: Synthesis Report. In Core Writing Team , H. Lee , & J. Romero (Eds.), Contribution of Working Groups I, II and III to the Sixth Assessment Report of the Intergovernmental Panel on Climate Change. IPCC. (P. Arias, M. Bustamante, I. Elgizouli, G. Flato, M. Howden, C. Méndez‐Vallejo, J. J. Pereira, R. Pichs‐Madruga, S. K. Rose, Y. Saheb, R. Sánchez Rodríguez, D. Ürge‐Vorsatz, C. Xiao, N. Yassaa, J. Romero, J. Kim, E. F. Haites, Y. Jung, R. Stavins, … C. Péan, Eds.). Intergovernmental Panel on Climate Change (IPCC). 10.59327/ipcc/ar6-9789291691647

[jane70040-bib-0022] Chakravarty, P. , Cozzi, G. , Ozgul, A. , & Aminian, K. (2019). A novel biomechanical approach for animal behaviour recognition using accelerometers. Methods in Ecology and Evolution, 10(6), 802–814. 10.1111/2041-210X.13172

[jane70040-bib-0023] Chambault, P. , Kovacs, K. M. , Lydersen, C. , Shpak, O. , Teilmann, J. , Albertsen, C. M. , & Heide‐Jørgensen, M. P. (2022). Future seasonal changes in habitat for Arctic whales during predicted ocean warming. Science Advances, 8(29), eabn2422. 10.1126/sciadv.abn2422 35867786 PMC9307241

[jane70040-bib-0024] Chan, W.‐P. , Lenoir, J. , Mai, G.‐S. , Kuo, H.‐C. , Chen, I.‐C. , & Shen, S.‐F. (2024). Climate velocities and species tracking in global mountain regions. Nature, 629(8010), 114–120. 10.1038/s41586-024-07264-9 38538797 PMC11062926

[jane70040-bib-0025] Chatterjee, N. , Wolfson, D. , Kim, D. , Velez, J. , Freeman, S. , Bacheler, N. M. , Shertzer, K. , Taylor, J. C. , & Fieberg, J. (2024). Modelling individual variability in habitat selection and movement using integrated step‐selection analysis. Methods in Ecology and Evolution, 15(6), 1034–1047. 10.1111/2041-210X.14321

[jane70040-bib-0026] Chow, P. K. Y. , Uchida, K. , & Koizumi, I. (2024). ‘Ripple effects’ of urban environmental characteristics on cognitive performances in Eurasian red squirrels. Journal of Animal Ecology, 93(8), 1078–1096. 10.1111/1365-2656.14126 38924529

[jane70040-bib-0027] Clavel, J. , Julliard, R. , & Devictor, V. (2011). Worldwide decline of specialist species: Toward a global functional homogenization? Frontiers in Ecology and the Environment, 9(4), 222–228. 10.1890/080216

[jane70040-bib-0028] Costa‐Pereira, R. , Moll, R. J. , Jesmer, B. R. , & Jetz, W. (2022). Animal tracking moves community ecology: Opportunities and challenges. Journal of Animal Ecology, 91(7), 1334–1344. 10.1111/1365-2656.13698 35388473 PMC10286655

[jane70040-bib-0029] Courbin, N. , Garel, M. , Marchand, P. , Duparc, A. , Debeffe, L. , Börger, L. , & Loison, A. (2022). Interacting lethal and nonlethal human activities shape complex risk tolerance behaviors in a mountain herbivore. Ecological Applications, 32(7), e2640. 10.1002/eap.2640 35443100 PMC13425691

[jane70040-bib-0030] Cowie, R. H. , Bouchet, P. , & Fontaine, B. (2022). The sixth mass extinction: Fact, fiction or speculation? Biological Reviews, 97(2), 640–663. 10.1111/brv.12816 35014169 PMC9786292

[jane70040-bib-0031] Crane, M. , Silva, I. , Marshall, B. M. , & Strine, C. T. (2021). Lots of movement, little progress: A review of reptile home range literature. PeerJ, 9, e11742. 10.7717/peerj.11742 34322323 PMC8300531

[jane70040-bib-0032] Davidson, S. C. , Cagnacci, F. , Newman, P. , Dettki, H. , Urbano, F. , Desmet, P. , Bajona, L. , Bryant, E. , Carneiro, A. P. B. , Dias, M. P. , Fujioka, E. , Gambin, D. , Hoenner, X. , Hunter, C. , Kato, A. , Kot, C. Y. , Kranstauber, B. , Lam, C. H. , Lepage, D. , … Rutz, C. (2025). Establishing bio‐logging data collections as dynamic archives of animal life on earth. Nature Ecology & Evolution, 9(2), 204–213. 10.1038/s41559-024-02585-4 39753915

[jane70040-bib-0033] Davoli, M. , Monsarrat, S. , Pedersen, R. Ø. , Scussolini, P. , Karger, D. N. , Normand, S. , & Svenning, J.‐C. (2024). Megafauna diversity and functional declines in Europe from the last interglacial to the present. Global Ecology and Biogeography, 33(1), 34–47. 10.1111/geb.13778

[jane70040-bib-0034] Delsink, A. , Vanak, A. T. , Ferreira, S. , & Slotow, R. (2013). Biologically relevant scales in large mammal management policies. Biological Conservation, 167, 116–126. 10.1016/j.biocon.2013.07.035

[jane70040-bib-0035] Dennhardt, A. J. , Duerr, A. E. , Brandes, D. , & Katzner, T. E. (2015). Integrating citizen‐science data with movement models to estimate the size of a migratory golden eagle population. Biological Conservation, 184, 68–78. 10.1016/j.biocon.2015.01.003

[jane70040-bib-0036] Dickie, M. , Serrouya, R. , McNay, R. S. , & Boutin, S. (2017). Faster and farther: Wolf movement on linear features and implications for hunting behaviour. Journal of Applied Ecology, 54(1), 253–263. 10.1111/1365-2664.12732

[jane70040-bib-0037] Ditmer, M. A. , Stoner, D. C. , Francis, C. D. , Barber, J. R. , Forester, J. D. , Choate, D. M. , Ironside, K. E. , Longshore, K. M. , Hersey, K. R. , Larsen, R. T. , McMillan, B. R. , Olson, D. D. , Andreasen, A. M. , Beckmann, J. P. , Holton, P. B. , Messmer, T. A. , & Carter, N. H. (2021). Artificial nightlight alters the predator–prey dynamics of an apex carnivore. Ecography, 44(2), 149–161. 10.1111/ecog.05251

[jane70040-bib-0038] Doherty, T. S. , Hays, G. C. , & Driscoll, D. A. (2021). Human disturbance causes widespread disruption of animal movement. Nature Ecology & Evolution, 5(4), 513–519. 10.1038/s41559-020-01380-1 33526889

[jane70040-bib-0039] Dombrovski, V. C. , Zhurauliou, D. V. , & Ashton‐Butt, A. (2022). Long‐term effects of rewilding on species composition: 22 years of raptor monitoring in the Chernobyl exclusion zone. Restoration Ecology, 30(8), e13633. 10.1111/rec.13633

[jane70040-bib-0040] Dominoni, D. M. , Halfwerk, W. , Baird, E. , Buxton, R. T. , Fernández‐Juricic, E. , Fristrup, K. M. , McKenna, M. F. , Mennitt, D. J. , Perkin, E. K. , Seymoure, B. M. , Stoner, D. C. , Tennessen, J. B. , Toth, C. A. , Tyrrell, L. P. , Wilson, A. , Francis, C. D. , Carter, N. H. , & Barber, J. R. (2020). Why conservation biology can benefit from sensory ecology. Nature Ecology & Evolution, 4(4), 502–511. 10.1038/s41559-020-1135-4 32203474

[jane70040-bib-0041] Ducatez, S. , Sayol, F. , Sol, D. , & Lefebvre, L. (2018). Are urban Vertebrates City specialists, artificial habitat exploiters, or environmental generalists? Integrative and Comparative Biology, 58(5), 929–938. 10.1093/icb/icy101 30102409

[jane70040-bib-0042] Dusenbery, D. B. (1992). Sensory ecology: How organisms acquire and respond to information. W.H. Freeman.

[jane70040-bib-0043] Elith, J. , & Leathwick, J. R. (2009). Species distribution models: Ecological explanation and prediction across space and time. Annual Review of Ecology, Evolution, and Systematics, 40, 677–697. 10.1146/annurev.ecolsys.110308.120159

[jane70040-bib-0044] Ellis‐Soto, D. , Oliver, R. Y. , Brum‐Bastos, V. , Demšar, U. , Jesmer, B. , Long, J. A. , Cagnacci, F. , Ossi, F. , Queiroz, N. , Hindell, M. , Kays, R. , Loretto, M.‐C. , Mueller, T. , Patchett, R. , Sims, D. W. , Tucker, M. A. , Ropert‐Coudert, Y. , Rutz, C. , & Jetz, W. (2023). A vision for incorporating human mobility in the study of human–wildlife interactions. Nature Ecology & Evolution, 7(9), 1362–1372. 10.1038/s41559-023-02125-6 37550509

[jane70040-bib-0046] Fieberg, J. , Freeman, S. , & Signer, J. (2024). Using lineups to evaluate goodness of fit of animal movement models. Methods in Ecology and Evolution, 15(6), 1048–1059. 10.1111/2041-210X.14336

[jane70040-bib-0047] Fieberg, J. R. , Forester, J. D. , Street, G. M. , Johnson, D. H. , ArchMiller, A. A. , & Matthiopoulos, J. (2018). Used‐habitat calibration plots: A new procedure for validating species distribution, resource selection, and step‐selection models. Ecography, 41(5), 737–752. 10.1111/ecog.03123

[jane70040-bib-0048] Forman, R. T. T. , & Alexander, L. E. (1998). Roads and their major ecological effects. Annual Review of Ecology, Evolution, and Systematics, 29, 207–231. 10.1146/annurev.ecolsys.29.1.207

[jane70040-bib-0049] Fortin, D. , Brooke, C. F. , Lamirande, P. , Fritz, H. , McLoughlin, P. D. , & Pays, O. (2020). Quantitative spatial ecology to promote human‐wildlife coexistence: A tool for integrated landscape management. Frontiers in Sustainable Food Systems, 4, 600363. 10.3389/fsufs.2020.600363

[jane70040-bib-0050] Fraser, K. C. , Davies, K. T. A. , Davy, C. M. , Ford, A. T. , Flockhart, D. T. T. , & Martins, E. G. (2018). Tracking the conservation promise of movement ecology. Frontiers in Ecology and Evolution, 6, 150. 10.3389/fevo.2018.00150

[jane70040-bib-0051] Fuchs, Y. H. , Edgar, G. , Bates, A. , Waldock, C. , & Smith, R. S. (2024). Limited net poleward movement amongst Australian reef species over a decade of climate extremes. Nature Climate Change, 14, 1087–1092. 10.1038/s41558-024-02116-w

[jane70040-bib-0052] Fuentes, M. , Van Doren, B. M. , Fink, D. , & Sheldon, D. (2023). BirdFlow: Learning seasonal bird movements from eBird data. Methods in Ecology and Evolution, 14(3), 923–938. 10.1111/2041-210X.14052

[jane70040-bib-0053] Gable, T. D. , Johnson‐Bice, S. M. , Homkes, A. T. , Fieberg, J. , & Bump, J. K. (2023). Wolves alter the trajectory of forests by shaping the central place foraging behaviour of an ecosystem engineer. Proceedings of the Royal Society B: Biological Sciences, 290(2010), 20231377. 10.1098/rspb.2023.1377 PMC10645084 37935367

[jane70040-bib-0054] Gaynor, K. M. , Abrahms, B. , Manlove, K. R. , Oestreich, W. K. , & Smith, J. A. (2024). Anthropogenic impacts at the interface of animal spatial and social behaviour. Philosophical Transactions of the Royal Society, B: Biological Sciences, 379(1912), 20220527. 10.1098/rstb.2022.0527 PMC11449167 39230457

[jane70040-bib-0055] Gaynor, K. M. , Fiorella, K. J. , Gregory, G. H. , Kurz, D. J. , Seto, K. L. , Withey, L. S. , & Brashares, J. S. (2016). War and wildlife: Linking armed conflict to conservation. Frontiers in Ecology and the Environment, 14(10), 533–542. 10.1002/fee.1433 PMC7753333 33362436

[jane70040-bib-0056] Gilbert, N. I. , Correia, R. A. , Silva, J. P. , Pacheco, C. , Catry, I. , Atkinson, P. W. , Gill, J. A. , & Franco, A. M. A. (2016). Are white storks addicted to junk food? Impacts of landfill use on the movement and behaviour of resident white storks (*Ciconia ciconia*) from a partially migratory population. Movement Ecology, 4(1), 7. 10.1186/s40462-016-0070-0 26981250 PMC4791752

[jane70040-bib-0057] Giroux, A. , Ortega, Z. , Attias, N. , Desbiez, A. L. J. , Valle, D. , Börger, L. , & Rodrigues Oliveira‐Santos, L. G. (2023). Activity modulation and selection for forests help giant anteaters to cope with temperature changes. Animal Behaviour, 201, 191–209. 10.1016/j.anbehav.2023.04.008

[jane70040-bib-0058] Griffin, A. S. , Netto, K. , & Peneaux, C. (2017). Neophilia, innovation and learning in an urbanized world: A critical evaluation of mixed findings. Current Opinion in Behavioral Sciences, 16, 15–22. 10.1016/j.cobeha.2017.01.004

[jane70040-bib-0059] Guerra, A. S. , Bui, A. , Klope, M. , Orr, D. A. , Shaffer, S. A. , & Young, H. S. (2022). Leaving more than footprints: Anthropogenic nutrient subsidies to a protected area. Ecosphere, 13(12), e4371. 10.1002/ecs2.4371

[jane70040-bib-0060] Gutmann Roberts, C. , Hindes, A. M. , & Britton, J. R. (2019). Factors influencing individual movements and behaviours of invasive European barbel *Barbus barbus* in a regulated river. Hydrobiologia, 830(1), 213–228. 10.1007/s10750-018-3864-9

[jane70040-bib-0061] Hale, R. , & Swearer, S. E. (2016). Ecological traps: Current evidence and future directions. Proceedings of the Royal Society B: Biological Sciences, 283(1824), 20152647. 10.1098/rspb.2015.2647 PMC4760169 26865295

[jane70040-bib-0062] Halfwerk, W. , & Slabbekoorn, H. (2015). Pollution going multimodal: The complex impact of the human‐altered sensory environment on animal perception and performance. Biology Letters, 11(4), 20141051. 10.1098/rsbl.2014.1051 25904319 PMC4424613

[jane70040-bib-0063] Harju, S. , Coates, P. , Dettenmaier, S. , Dinkins, J. , Jackson, P. , & Chenaille, M. (2021). Estimating trends of common raven populations in North America, 1966–2018. Human‐Wildlife Interactions, 15(3), 248–269. 10.26077/c27f-e335

[jane70040-bib-0064] Harris, J. , Bullock, J. , Pettorelli, N. , Perring, M. , & Mercer, T. (2024). Novel ecosystems: The new normal? https://www.britishecologicalsociety.org/novel‐ecosystems‐the‐new‐normal/

[jane70040-bib-0065] Hastie, G. D. , Lepper, P. , McKnight, J. C. , Milne, R. , Russell, D. J. F. , & Thompson, D. (2021). Acoustic risk balancing by marine mammals: Anthropogenic noise can influence the foraging decisions by seals. Journal of Applied Ecology, 58(9), 1854–1863. 10.1111/1365-2664.13931

[jane70040-bib-0066] Hawkes, C. (2009). Linking movement behaviour, dispersal and population processes: Is individual variation a key? Journal of Animal Ecology, 78(5), 894–906. 10.1111/j.1365-2656.2009.01534.x 19302396

[jane70040-bib-0067] Hays, G. C. , Bailey, H. , Bograd, S. J. , Bowen, W. D. , Campagna, C. , Carmichael, R. H. , Casale, P. , Chiaradia, A. , Costa, D. P. , Cuevas, E. , de Bruyn, P. J. N. , Dias, M. P. , Duarte, C. M. , Dunn, D. C. , Dutton, P. H. , Esteban, N. , Friedlaender, A. , Goetz, K. T. , Godley, B. J. , … Sequeira, A. M. M. (2019). Translating marine animal tracking data into conservation policy and management. Trends in Ecology & Evolution, 34(5), 459–473. 10.1016/j.tree.2019.01.009 30879872

[jane70040-bib-0068] Hebblewhite, M. , & Merrill, E. H. (2009). Trade‐offs between predation risk and forage differ between migrant strategies in a migratory ungulate. Ecology, 90(12), 3445–3454. 10.1890/08-2090.1 20120812

[jane70040-bib-0069] Herbert‐Read, J. E. , Kremer, L. , Bruintjes, R. , Radford, A. N. , & Ioannou, C. C. (2017). Anthropogenic noise pollution from pile‐driving disrupts the structure and dynamics of fish shoals. Proceedings of the Royal Society B: Biological Sciences, 284(1863), 20171627. 10.1098/rspb.2017.1627 PMC5627215 28954915

[jane70040-bib-0070] Hertel, A. G. , Niemelä, P. T. , Dingemanse, N. J. , & Mueller, T. (2020). A guide for studying among‐individual behavioral variation from movement data in the wild. Movement Ecology, 8(1), 30. 10.1186/s40462-020-00216-8 32612837 PMC7325061

[jane70040-bib-0071] Hobbs, R. J. , Arico, S. , Aronson, J. , Baron, J. S. , Bridgewater, P. , Cramer, V. A. , Epstein, P. R. , Ewel, J. J. , Klink, C. A. , Lugo, A. E. , Norton, D. , Ojima, D. , Richardson, D. M. , Sanderson, E. W. , Valladares, F. , Vilà, M. , Zamora, R. , & Zobel, M. (2006). Novel ecosystems: Theoretical and management aspects of the new ecological world order. Global Ecology and Biogeography, 15(1), 1–7. 10.1111/j.1466-822X.2006.00212.x

[jane70040-bib-0072] Hody, J. W. , & Kays, R. (2018). Mapping the expansion of coyotes (*Canis latrans*) across north and Central America. ZooKeys, 759, 81–97. 10.3897/zookeys.759.15149 PMC5974007 29861647

[jane70040-bib-0073] Hoekstra, B. , Bouten, W. , Dokter, A. , van Gasteren, H. , Turnhout, C. , Kranstauber, B. , van Loon, E. , Leijnse, H. , & Shamoun‐Baranes, J. (2024). Fireworks disturbance across bird communities. Frontiers in Ecology and the Environment, 22(1), e2694. 10.1002/fee.2694

[jane70040-bib-0074] Holton, M. D. , Wilson, R. P. , Teilmann, J. , & Siebert, U. (2021). Animal tag technology keeps coming of age: An engineering perspective. Philosophical Transactions of the Royal Society, B: Biological Sciences, 376(1831), 20200229. 10.1098/rstb.2020.0229 PMC8237169 34176328

[jane70040-bib-0075] Hussey, N. E. , Kessel, S. T. , Aarestrup, K. , Cooke, S. J. , Cowley, P. D. , Fisk, A. T. , Harcourt, R. G. , Holland, K. N. , Iverson, S. J. , Kocik, J. F. , Mills Flemming, J. E. , & Whoriskey, F. G. (2015). Aquatic animal telemetry: A panoramic window into the underwater world. Science, 348(6240), 1255642. 10.1126/science.1255642 26068859

[jane70040-bib-0076] Imlay, T. L. , Nickerson, D. , & Horn, A. G. (2019). Temperature and breeding success for cliff swallows (*Petrochelidon pyrrhonota*) nesting on man‐made structures: Ecological traps? Canadian Journal of Zoology, 97(5), 429–435. 10.1139/cjz-2018-0224

[jane70040-bib-0077] Inamine, H. , Miller, A. , Roxburgh, S. , Buckling, A. , & Shea, K. (2022). Pulse and press disturbances have different effects on transient community dynamics. The American Naturalist, 200(4), 571–583. 10.1086/720618 36150192

[jane70040-bib-0210] IPBES . (2019). Summary for policymakers of the global assessment report on biodiversity and ecosystem services (summary for policy makers). Zenodo. 10.5281/ZENODO.3553579

[jane70040-bib-0078] Johnson, C. J. , & St‐Laurent, M.‐H. (2011). Unifying framework for understanding impacts of human developments on wildlife. In D. E. Naugle (Ed.), Energy development and wildlife conservation in Western North America (pp. 27–54). Island Press/Center for Resource Economics. 10.5822/978-1-61091-022-4_3

[jane70040-bib-0079] Jones, P. F. , Jakes, A. F. , Vegter, S. E. , & Verhage, M. S. (2022). Is it the road or the fence? Influence of linear anthropogenic features on the movement and distribution of a partially migratory ungulate. Movement Ecology, 10(1), 37. 10.1186/s40462-022-00336-3 36038930 PMC9422137

[jane70040-bib-0080] Kadykalo, A. N. , Buxton, R. T. , Morrison, P. , Anderson, C. M. , Bickerton, H. , Francis, C. M. , Smith, A. C. , & Fahrig, L. (2021). Bridging research and practice in conservation. Conservation Biology, 35(6), 1725–1737. 10.1111/cobi.13732 33738830 PMC9291548

[jane70040-bib-0081] Katzner, T. E. , & Arlettaz, R. (2020). Evaluating Contributions of recent tracking‐based animal movement ecology to conservation management. Frontiers in Ecology and Evolution, 7, 519. 10.3389/fevo.2019.00519

[jane70040-bib-0082] Kays, R. , Crofoot, M. C. , Jetz, W. , & Wikelski, M. (2015). Terrestrial animal tracking as an eye on life and planet. Science, 348(6240), aaa2478. 10.1126/science.aaa2478 26068858

[jane70040-bib-0214] Kays, R. , Davidson, S. C. , Berger, M. , Bohrer, G. , Fiedler, W. , Flack, A. , Hirt, J. , Hahn, C. , Gauggel, D. , Russell, B. , Kölzsch, A. , Lohr, A. , Partecke, J. , Quetting, M. , Safi, K. , Scharf, A. , Schneider, G. , Lang, I. , Schaeuffelhut, F. , … Wikelski, M. (2021). The Movebank system for studying global animal movement and demography. Methods in Ecology and Evolution, 13(2), 419–431. 10.1111/2041-210x.13767

[jane70040-bib-0083] Kemppinen, J. , Lembrechts, J. J. , Van Meerbeek, K. , Carnicer, J. , Chardon, N. I. , Kardol, P. , Lenoir, J. , Liu, D. , Maclean, I. , Pergl, J. , Saccone, P. , Senior, R. A. , Shen, T. , Słowińska, S. , Vandvik, V. , von Oppen, J. , Aalto, J. , Ayalew, B. , Bates, O. , … De Frenne, P. (2024). Microclimate, an important part of ecology and biogeography. Global Ecology and Biogeography, 33(6), e13834. 10.1111/geb.13834

[jane70040-bib-0084] Klappstein, N. J. , Michelot, T. , Fieberg, J. , Pedersen, E. J. , & Mills Flemming, J. (2024). Step selection functions with non‐linear and random effects. Methods in Ecology and Evolution, 15(8), 1332–1346. 10.1111/2041-210X.14367

[jane70040-bib-0085] Klappstein, N. J. , Potts, J. R. , Michelot, T. , Börger, L. , Pilfold, N. W. , Lewis, M. A. , & Derocher, A. E. (2022). Energy‐based step selection analysis: Modelling the energetic drivers of animal movement and habitat use. Journal of Animal Ecology, 91(5), 946–957. 10.1111/1365-2656.13687 35277858

[jane70040-bib-0086] Kölzsch, A. , Lameris, T. K. , Müskens, G. J. D. M. , Schreven, K. H. T. , Buitendijk, N. H. , Kruckenberg, H. , Moonen, S. , Heinicke, T. , Cao, L. , Madsen, J. , Wikelski, M. , & Nolet, B. A. (2023). Wild goose chase: Geese flee high and far, and with aftereffects from new Year's fireworks. Conservation Letters, 16(1), e12927. 10.1111/conl.12927

[jane70040-bib-0087] Kristan, W. B. , & Boarman, W. I. (2007). Effects of anthropogenic developments on common raven nesting biology in the West Mojave Desert. Ecological Applications, 17(6), 1703–1713. 10.1890/06-1114.1 17913134

[jane70040-bib-0088] Leblond, M. , Dussault, C. , & Ouellet, J.‐.P. (2013). Avoidance of roads by large herbivores and its relation to disturbance intensity. Journal of Zoology, 289(1), 32–40. 10.1111/j.1469-7998.2012.00959.x

[jane70040-bib-0089] Lempidakis, E. , Shepard, E. L. C. , Ross, A. N. , Matsumoto, S. , Koyama, S. , Takeuchi, I. , & Yoda, K. (2022). Pelagic seabirds reduce risk by flying into the eye of the storm. Proceedings of the National Academy of Sciences of the United States of America, 119(41), e2212925119. 10.1073/pnas.2212925119 36194636 PMC9565516

[jane70040-bib-0090] Lesmerises, F. , Dussault, C. , & St‐Laurent, M.‐H. (2013). Major roadwork impacts the space use behaviour of gray wolf. Landscape and Urban Planning, 112, 18–25. 10.1016/j.landurbplan.2012.12.011

[jane70040-bib-0091] Levin, S. A. , & Paine, R. T. (1974). Disturbance, patch formation, and community structure. Proceedings of the National Academy of Sciences of the United States of America, 71(7), 2744–2747. 10.1073/pnas.71.7.2744 4527752 PMC388546

[jane70040-bib-0092] Loss, S. R. , Will, T. , Loss, S. S. , & Marra, P. P. (2014). Bird–building collisions in the United States: Estimates of annual mortality and species vulnerability. The Condor, 116(1), 8–23. 10.1650/CONDOR-13-090.1

[jane70040-bib-0093] Loss, S. R. , Will, T. , & Marra, P. P. (2013). Estimates of bird collision mortality at wind facilities in the contiguous United States. Biological Conservation, 168, 201–209. 10.1016/j.biocon.2013.10.007

[jane70040-bib-0094] Lustenhouwer, N. , Moerman, F. , Altermatt, F. , Bassar, R. D. , Bocedi, G. , Bonte, D. , Dey, S. , Fronhofer, E. A. , Da Rocha, É. G. , Giometto, A. , Lancaster, L. T. , Prather, R. B. , Saastamoinen, M. , Travis, J. M. J. , Urquhart, C. A. , Weiss‐Lehman, C. , Williams, J. L. , Börger, L. , & Berger, D. (2023). Experimental evolution of dispersal: Unifying theory, experiments and natural systems. Journal of Animal Ecology, 92(6), 1113–1123. 10.1111/1365-2656.13930 37087688

[jane70040-bib-0095] Ma, D. , Abrahms, B. , Allgeier, J. , Newbold, T. , Weeks, B. C. , & Carter, N. H. (2024). Global expansion of human‐wildlife overlap in the 21st century. Science Advances, 10(34), eadp7706. 10.1126/sciadv.adp7706 39167651 PMC11338222

[jane70040-bib-0096] Macdonald, D. W. , & Johnson, D. D. P. (2015). Patchwork planet: The resource dispersion hypothesis, society, and the ecology of life. Journal of Zoology, 295(2), 75–107. 10.1111/jzo.12202

[jane70040-bib-0097] Maes, S. L. , Perring, M. P. , Cohen, R. , Akinnifesi, F. K. , Bargués‐Tobella, A. , Bastin, J.‐F. , Bauters, M. , Bernardino, P. N. , Brancalion, P. H. S. , Bullock, J. M. , Ellison, D. , Fayolle, A. , Fremout, T. , Gann, G. D. , Hishe, H. , Holmgren, M. , Ilstedt, U. , Mahy, G. , Messier, C. , … Muys, B. (2024). Explore before you restore: Incorporating complex systems thinking in ecosystem restoration. Journal of Applied Ecology, 61(5), 922–939. 10.1111/1365-2664.14614

[jane70040-bib-0211] Malishev, M. , & Kramer‐Schadt, S. (2021). Movement, models, and metabolism: Individual‐based energy budget models as next‐generation extensions for predicting animal movement outcomes across scales. Ecological Modelling, 441, 109413. 10.1016/j.ecolmodel.2020.109413

[jane70040-bib-0098] Manly, B. F. J. , McDonald, L. L. , & Thomas, T. (2002). Review of resource selection by animals: Statistical design and analysis for field studies ‐ second edition. Journal of Animal Ecology, 63(3), 745–746. 10.2307/5247

[jane70040-bib-0099] Månsson, J. , Eriksson, L. , Hodgson, I. , Elmberg, J. , Bunnefeld, N. , Hessel, R. , Johansson, M. , Liljebäck, N. , Nilsson, L. , Olsson, C. , Pärt, T. , Sandström, C. , Tombre, I. , & Redpath, S. M. (2023). Understanding and overcoming obstacles in adaptive management. Trends in Ecology & Evolution, 38(1), 55–71. 10.1016/j.tree.2022.08.009 36202636

[jane70040-bib-0100] Marshall, B. M. , Crane, M. , Silva, I. , Strine, C. T. , Jones, M. D. , Hodges, C. W. , Suwanwaree, P. , Artchawakom, T. , Waengsothorn, S. , & Goode, M. (2020). No room to roam: King cobras reduce movement in agriculture. Movement Ecology, 8(1), 33. 10.1186/s40462-020-00219-5 32774861 PMC7397683

[jane70040-bib-0101] Masden, E. A. , Haydon, D. T. , Fox, A. D. , Furness, R. W. , Bullman, R. , & Desholm, M. (2009). Barriers to movement: Impacts of wind farms on migrating birds. ICES Journal of Marine Science, 66(4), 746–753. 10.1093/icesjms/fsp031

[jane70040-bib-0102] Matthiopoulos, J. (2003). The use of space by animals as a function of accessibility and preference. Ecological Modelling, 159(2), 239–268. 10.1016/S0304-3800(02)00293-4

[jane70040-bib-0103] Matthiopoulos, J. (2022). Defining, estimating, and understanding the fundamental niches of complex animals in heterogeneous environments. Ecological Monographs, 92(4), e1545. 10.1002/ecm.1545

[jane70040-bib-0104] Matthiopoulos, J. , Hebblewhite, M. , Aarts, G. , & Fieberg, J. (2011). Generalized functional responses for species distributions. Ecology, 92(3), 583–589. 10.1890/10-0751.1 21608467

[jane70040-bib-0105] McClintock, B. T. , & Michelot, T. (2018). momentuHMM: R package for generalized hidden Markov models of animal movement. Methods in Ecology and Evolution, 9(6), 1518–1530. 10.1111/2041-210X.12995

[jane70040-bib-0106] McCrea, R. , King, R. , Graham, L. , & Börger, L. (2023). Realising the promise of large data and complex models. Methods in Ecology and Evolution, 14(1), 4–11. 10.1111/2041-210X.14050

[jane70040-bib-0107] McGowan, J. , Beger, M. , Lewison, R. L. , Harcourt, R. , Campbell, H. , Priest, M. , Dwyer, R. G. , Lin, H.‐Y. , Lentini, P. , Dudgeon, C. , McMahon, C. , Watts, M. , & Possingham, H. P. (2017). Integrating research using animal‐borne telemetry with the needs of conservation management. Journal of Applied Ecology, 54(2), 423–429. 10.1111/1365-2664.12755

[jane70040-bib-0108] McKinney, M. L. (2006). Urbanization as a major cause of biotic homogenization. Biological Conservation, 127(3), 247–260. 10.1016/j.biocon.2005.09.005

[jane70040-bib-0109] Meisingset, E. L. , Loe, L. E. , Brekkum, Ø. , Bischof, R. , Rivrud, I. M. , Lande, U. S. , Zimmermann, B. , Veiberg, V. , & Mysterud, A. (2018). Spatial mismatch between management units and movement ecology of a partially migratory ungulate. Journal of Applied Ecology, 55(2), 745–753. 10.1111/1365-2664.13003

[jane70040-bib-0110] Mensinger, M. A. , Hawkes, J. P. , Goulette, G. S. , Mortelliti, A. , Blomberg, E. J. , & Zydlewski, J. D. (2024). Dams facilitate predation during Atlantic salmon (*Salmo salar*) smolt migration. Canadian Journal of Fisheries and Aquatic Sciences, 81(1), 38–51. 10.1139/cjfas-2023-0175

[jane70040-bib-0111] Merkle, J. A. , Potts, J. R. , & Fortin, D. (2017). Energy benefits and emergent space use patterns of an empirically parameterized model of memory‐based patch selection. Oikos, 126(2), 185–195. 10.1111/oik.03356

[jane70040-bib-0112] Michelot, T. , Blackwell, P. G. , Chamaillé‐Jammes, S. , & Matthiopoulos, J. (2020). Inference in MCMC step selection models. Biometrics, 76(2), 438–447. 10.1111/biom.13170 31654395

[jane70040-bib-0113] Michelot, T. , Blackwell, P. G. , & Matthiopoulos, J. (2019). Linking resource selection and step selection models for habitat preferences in animals. Ecology, 100(1), e02452. 10.1002/ecy.2452 30047993

[jane70040-bib-0114] Michelot, T. , Gloaguen, P. , Blackwell, P. G. , & Étienne, M.‐P. (2019). The Langevin diffusion as a continuous‐time model of animal movement and habitat selection. Methods in Ecology and Evolution, 10(11), 1894–1907. 10.1111/2041-210X.13275

[jane70040-bib-0115] Milner, J. E. , Blackwell, P. G. , & Niu, M. (2021). Modelling and inference for the movement of interacting animals. Methods in Ecology and Evolution, 12(1), 54–69. 10.1111/2041-210X.13468

[jane70040-bib-0116] Moorcroft, P. R. , Lewis, M. A. , & Crabtree, R. L. (2006). Mechanistic home range models capture spatial patterns and dynamics of coyote territories in Yellowstone. Proceedings of the Royal Society B: Biological Sciences, 273(1594), 1651–1659. 10.1098/rspb.2005.3439 PMC1704082 16769637

[jane70040-bib-0117] Muff, S. , Signer, J. , & Fieberg, J. (2020). Accounting for individual‐specific variation in habitat‐selection studies: Efficient estimation of mixed‐effects models using Bayesian or frequentist computation. Journal of Animal Ecology, 89(1), 80–92. 10.1111/1365-2656.13087 31454066

[jane70040-bib-0118] Nathan, R. , Getz, W. M. , Revilla, E. , Holyoak, M. , Kadmon, R. , Saltz, D. , & Smouse, P. E. (2008). A movement ecology paradigm for unifying organismal movement research. Proceedings of the National Academy of Sciences of the United States of America, 105(49), 19052–19059. 10.1073/pnas.0800375105 19060196 PMC2614714

[jane70040-bib-0119] Nathan, R. , Monk, C. T. , Arlinghaus, R. , Adam, T. , Alós, J. , Assaf, M. , Baktoft, H. , Beardsworth, C. E. , Bertram, M. G. , Bijleveld, A. I. , Brodin, T. , Brooks, J. L. , Campos‐Candela, A. , Cooke, S. J. , Gjelland, K. Ø. , Gupte, P. R. , Harel, R. , Hellström, G. , Jeltsch, F. , … Jarić, I. (2022). Big‐data approaches lead to an increased understanding of the ecology of animal movement. Science, 375(6582), eabg1780. 10.1126/science.abg1780 35175823

[jane70040-bib-0120] Newbold, T. , Hudson, L. N. , Hill, S. L. L. , Contu, S. , Lysenko, I. , Senior, R. A. , Börger, L. , Bennett, D. J. , Choimes, A. , Collen, B. , Day, J. , De Palma, A. , Díaz, S. , Echeverria‐Londoño, S. , Edgar, M. J. , Feldman, A. , Garon, M. , Harrison, M. L. K. , Alhusseini, T. , … Purvis, A. (2015). Global effects of land use on local terrestrial biodiversity. Nature, 520(7545), 45–50. 10.1038/nature14324 25832402

[jane70040-bib-0121] Newman, K. , King, R. , Elvira, V. , de Valpine, P. , McCrea, R. S. , & Morgan, B. J. T. (2023). State‐space models for ecological time‐series data: Practical model‐fitting. Methods in Ecology and Evolution, 14(1), 26–42. 10.1111/2041-210X.13833

[jane70040-bib-0122] Newman, R. , & Noy, I. (2023). The global costs of extreme weather that are attributable to climate change. Nature Communications, 14(1), 6103. 10.1038/s41467-023-41888-1 PMC10541421 37775690

[jane70040-bib-0123] Newsome, S. D. , Ralls, K. , Van Horn Job, C. , Fogel, M. L. , & Cypher, B. L. (2010). Stable isotopes evaluate exploitation of anthropogenic foods by the endangered San Joaquin kit fox (*Vulpes macrotis mutica*). Journal of Mammalogy, 91(6), 1313–1321. 10.1644/09-MAMM-A-362.1

[jane70040-bib-0124] Niebuhr, B. B. , Sant'Ana, D. , Panzacchi, M. , van Moorter, B. , Sandström, P. , Morato, R. G. , & Skarin, A. (2022). Renewable energy infrastructure impacts biodiversity beyond the area it occupies. Proceedings of the National Academy of Sciences of the United States of America, 119(48), e2208815119. 10.1073/pnas.2208815119 36409906 PMC9860302

[jane70040-bib-0125] Nimmo, D. G. , Avitabile, S. , Banks, S. C. , Bliege Bird, R. , Callister, K. , Clarke, M. F. , Dickman, C. R. , Doherty, T. S. , Driscoll, D. A. , Greenville, A. C. , Haslem, A. , Kelly, L. T. , Kenny, S. A. , Lahoz‐Monfort, J. J. , Lee, C. , Leonard, S. , Moore, H. , Newsome, T. M. , Parr, C. L. , … Bennett, A. F. (2019). Animal movements in fire‐prone landscapes. Biological Reviews, 94(3), 981–998. 10.1111/brv.12486 30565370

[jane70040-bib-0126] Nisi, A. C. , Benson, J. F. , & Wilmers, C. C. (2022). Puma responses to unreliable human cues suggest an ecological trap in a fragmented landscape. Oikos, 2022(5), e09051. 10.1111/oik.09051

[jane70040-bib-0127] Niu, M. , Blackwell, P. G. , & Skarin, A. (2016). Modeling interdependent animal movement in continuous time. Biometrics, 72(2), 315–324. 10.1111/biom.12454 26812666

[jane70040-bib-0128] Nuijten, R. J. M. , Katzner, T. E. , Allen, A. M. , Bijleveld, A. I. , Boorsma, T. , Börger, L. , Cagnacci, F. , Hart, T. , Henley, M. A. , Herren, R. M. , Kok, E. M. A. , Maree, B. , Nebe, B. , Shohami, D. , Vogel, S. M. , Walker, P. , Heitkönig, I. M. A. , & Milner‐Gulland, E. J. (2023). Priorities for translating goodwill between movement ecologists and conservation practitioners into effective collaboration. Conservation Science and Practice, 5(1), e12870. 10.1111/csp2.12870

[jane70040-bib-0129] Oliver, R. , Chapman, M. , Ellis‐Soto, D. , Brum‐Bastos, V. , Cagnacci, F. , Long, J. , Loretto, M.‐C. , Patchett, R. B. , & Rutz, C. (2024). Access to human‐mobility data is essential for building a sustainable future. Cell Reports Sustainability, 1(4), 100077. 10.1016/j.crsus.2024.100077

[jane70040-bib-0130] Oro, D. , Genovart, M. , Tavecchia, G. , Fowler, M. S. , & Martínez‐Abraín, A. (2013). Ecological and evolutionary implications of food subsidies from humans. Ecology Letters, 16(12), 1501–1514. 10.1111/ele.12187 24134225

[jane70040-bib-0131] Owen, M. A. , Swaisgood, R. R. , & Blumstein, D. T. (2017). Contextual influences on animal decision‐making: Significance for behavior‐based wildlife conservation and management. Integrative Zoology, 12(1), 32–48. 10.1111/1749-4877.12235 27605354

[jane70040-bib-0132] Patterson, T. A. , Thomas, L. , Wilcox, C. , Ovaskainen, O. , & Matthiopoulos, J. (2008). State–space models of individual animal movement. Trends in Ecology & Evolution, 23(2), 87–94. 10.1016/j.tree.2007.10.009 18191283

[jane70040-bib-0208] Pereira, H. M. , Martins, I. S. , Rosa, I. M. D. , Kim, H. , Leadley, P. , Popp, A. , van Vuuren, D. P. , Hurtt, G. , Quoss, L. , Arneth, A. , Baisero, D. , Bakkenes, M. , Chaplin‐Kramer, R. , Chini, L. , Di Marco, M. , Ferrier, S. , Fujimori, S. , Guerra, C. A. , Harfoot, M. , … Alkemade, R. (2024). Global trends and scenarios for terrestrial biodiversity and ecosystem services from 1900 to 2050. Science, 384(6694), 458–465. 10.1126/science.adn3441 38662818

[jane70040-bib-0133] Perona, A. M. , Urios, V. , & López‐López, P. (2019). Holidays? Not for all. Eagles have larger home ranges on holidays as a consequence of human disturbance. Biological Conservation, 231, 59–66. 10.1016/j.biocon.2019.01.010

[jane70040-bib-0134] Pfaller, J. B. , Goforth, K. M. , Gil, M. A. , Savoca, M. S. , & Lohmann, K. J. (2020). Odors from marine plastic debris elicit foraging behavior in sea turtles. Current Biology, 30(5), R213–R214. 10.1016/j.cub.2020.01.071 32155421

[jane70040-bib-0135] Plummer, K. E. , Hale, J. D. , O'Callaghan, M. J. , Sadler, J. P. , & Siriwardena, G. M. (2016). Investigating the impact of street lighting changes on garden moth communities. Journal of Urban Ecology, 2(1), juw004. 10.1093/jue/juw004

[jane70040-bib-0136] Poloczanska, E. S. , Brown, C. J. , Sydeman, W. J. , Kiessling, W. , Schoeman, D. S. , Moore, P. J. , Brander, K. , Bruno, J. F. , Buckley, L. B. , Burrows, M. T. , Duarte, C. M. , Halpern, B. S. , Holding, J. , Kappel, C. V. , O'Connor, M. I. , Pandolfi, J. M. , Parmesan, C. , Schwing, F. , Thompson, S. A. , & Richardson, A. J. (2013). Global imprint of climate change on marine life. Nature Climate Change, 3(10), 919–925. 10.1038/nclimate1958

[jane70040-bib-0137] Potts, J. R. , & Börger, L. (2023). How to scale up from animal movement decisions to spatiotemporal patterns: An approach via step selection. Journal of Animal Ecology, 92(1), 16–29. 10.1111/1365-2656.13832 36321473 PMC10099581

[jane70040-bib-0138] Potts, J. R. , Börger, L. , Strickland, B. K. , & Street, G. M. (2022). Assessing the predictive power of step selection functions: How social and environmental interactions affect animal space use. Methods in Ecology and Evolution, 13(8), 1805–1818. 10.1111/2041-210X.13904

[jane70040-bib-0139] Potts, J. R. , & Lewis, M. A. (2014). How do animal territories form and change? Lessons from 20 years of mechanistic modelling. Proceedings of the Royal Society B: Biological Sciences, 281(1784), 20140231. 10.1098/rspb.2014.0231 PMC4043092 24741017

[jane70040-bib-0140] Potts, J. R. , & Schlägel, U. E. (2020). Parametrizing diffusion‐taxis equations from animal movement trajectories using step selection analysis. Methods in Ecology and Evolution, 11(9), 1092–1105. 10.1111/2041-210X.13406

[jane70040-bib-0141] Ranc, N. , Moorcroft, P. R. , Ossi, F. , & Cagnacci, F. (2021). Experimental evidence of memory‐based foraging decisions in a large wild mammal. Proceedings of the National Academy of Sciences of the United States of America, 118(15), e2014856118. 10.1073/pnas.2014856118 33837149 PMC8053919

[jane70040-bib-0213] Ratnayake, M. N. , Amarathunga, D. C. , Zaman, A. , Dyer, A. G. , & Dorin, A. (2022). Spatial monitoring and insect behavioural analysis using computer vision for precision pollination. International Journal of Computer Vision, 131(3), 591–606. 10.1007/s11263-022-01715-4

[jane70040-bib-0142] Richardson, K. , Steffen, W. , Lucht, W. , Bendtsen, J. , Cornell, S. E. , Donges, J. F. , Drüke, M. , Fetzer, I. , Bala, G. , von Bloh, W. , Feulner, G. , Fiedler, S. , Gerten, D. , Gleeson, T. , Hofmann, M. , Huiskamp, W. , Kummu, M. , Mohan, C. , Nogués‐Bravo, D. , … Rockström, J. (2023). Earth beyond six of nine planetary boundaries. Science Advances, 9(37), eadh2458. 10.1126/sciadv.adh2458 37703365 PMC10499318

[jane70040-bib-0143] Rieber, C. J. , Hefley, T. J. , & Haukos, D. A. (2024). Treed gaussian processes for animal movement modeling. Ecology and Evolution, 14(6), e11447. 10.1002/ece3.11447 38832142 PMC11144715

[jane70040-bib-0144] Rilov, G. , Canning‐Clode, J. , & Guy‐Haim, T. (2024). Ecological impacts of invasive ecosystem engineers: A global perspective across terrestrial and aquatic systems. Functional Ecology, 38(1), 37–51. 10.1111/1365-2435.14406

[jane70040-bib-0145] Rinella, M. J. , Strong, D. J. , & Vermeire, L. T. (2020). Omitted variable bias in studies of plant interactions. Ecology, 101(6), e03020. 10.1002/ecy.3020 32083313

[jane70040-bib-0146] Riotte‐Lambert, L. , & Matthiopoulos, J. (2020). Environmental predictability as a cause and consequence of animal movement. Trends in Ecology & Evolution, 35(2), 163–174. 10.1016/j.tree.2019.09.009 31699411

[jane70040-bib-0147] Rolland, R. M. , Parks, S. E. , Hunt, K. E. , Castellote, M. , Corkeron, P. J. , Nowacek, D. P. , Wasser, S. K. , & Kraus, S. D. (2012). Evidence that ship noise increases stress in right whales. Proceedings of the Royal Society B: Biological Sciences, 279(1737), 2363–2368. 10.1098/rspb.2011.2429 PMC3350670 22319129

[jane70040-bib-0148] Romano, M. D. , Piatt, J. F. , & Roby, D. D. (2006). Testing the junk‐food hypothesis on marine birds: Effects of prey type on growth and development. Waterbirds, 29(4), 407–414. 10.1675/1524-4695(2006)29[407:TTJHOM]2.0.CO;2

[jane70040-bib-0149] Rueda‐Uribe, C. , Herrera‐Alsina, L. , Lancaster, L. T. , Capellini, I. , Layton, K. K. S. , & Travis, J. M. J. (2024). Citizen science data reveal altitudinal movement and seasonal ecosystem use by hummingbirds in the Andes Mountains. Ecography, 2024(3), e06735. 10.1111/ecog.06735

[jane70040-bib-0150] Russell, C. J. G. , Franco, A. M. A. , Atkinson, P. W. , Väli, Ü. , & Ashton‐Butt, A. (2024). Active European warzone impacts raptor migration. Current Biology, 34(10), 2272–2277.e2. 10.1016/j.cub.2024.04.047 38772328

[jane70040-bib-0152] Rutz, C. (2022a). Register animal‐tracking tags to boost conservation. Nature, 609(7926), 221. 10.1038/d41586-022-02821-6 36071182

[jane70040-bib-0153] Rutz, C. (2022b). Studying pauses and pulses in human mobility and their environmental impacts. Nature Reviews Earth and Environment, 3(3), 157–159. 10.1038/s43017-022-00276-x

[jane70040-bib-0154] Rutz, C. , Loretto, M.‐C. , Bates, A. E. , Davidson, S. C. , Duarte, C. M. , Jetz, W. , Johnson, M. , Kato, A. , Kays, R. , Mueller, T. , Primack, R. B. , Ropert‐Coudert, Y. , Tucker, M. A. , Wikelski, M. , & Cagnacci, F. (2020). COVID‐19 lockdown allows researchers to quantify the effects of human activity on wildlife. Nature Ecology & Evolution, 4(9), 1156–1159. 10.1038/s41559-020-1237-z 32572222

[jane70040-bib-0155] Sarkar, R. , & Bhadra, A. (2022). How do animals navigate the urban jungle? A review of cognition in urban‐adapted animals. Current Opinion in Behavioral Sciences, 46, 101177. 10.1016/j.cobeha.2022.101177

[jane70040-bib-0156] Schaub, A. , Ostwald, J. , & Siemers, B. M. (2008). Foraging bats avoid noise. Journal of Experimental Biology, 211(19), 3174–3180. 10.1242/jeb.022863 18805817

[jane70040-bib-0157] Schlaepfer, M. A. , Runge, M. C. , & Sherman, P. W. (2002). Ecological and evolutionary traps. Trends in Ecology & Evolution, 17(10), 474–480. 10.1016/S0169-5347(02)02580-6

[jane70040-bib-0158] Schlägel, U. E. , Signer, J. , Herde, A. , Eden, S. , Jeltsch, F. , Eccard, J. A. , & Dammhahn, M. (2019). Estimating interactions between individuals from concurrent animal movements. Methods in Ecology and Evolution, 10(8), 1234–1245. 10.1111/2041-210X.13235

[jane70040-bib-0159] Schmitz, O. J. , Sylvén, M. , Atwood, T. B. , Bakker, E. S. , Berzaghi, F. , Brodie, J. F. , Cromsigt, J. P. G. M. , Davies, A. B. , Leroux, S. J. , Schepers, F. J. , Smith, F. A. , Stark, S. , Svenning, J.‐C. , Tilker, A. , & Ylänne, H. (2023). Trophic rewilding can expand natural climate solutions. Nature Climate Change, 13(4), 324–333. 10.1038/s41558-023-01631-6

[jane70040-bib-0160] Schoen, J. M. , DeFries, R. , & Cushman, S. (2025). Open‐source, environmentally dynamic machine learning models demonstrate behavior‐dependent utilization of mixed‐use landscapes by jaguars (*Panthera onca*). Biological Conservation, 302, 110978. 10.1016/j.biocon.2025.110978

[jane70040-bib-0161] Selier, J. , Slotow, R. , & Di Minin, E. (2015). Large mammal distribution in a Transfrontier landscape: Trade‐offs between resource availability and human disturbance. Biotropica, 47(3), 389–397. 10.1111/btp.12217

[jane70040-bib-0162] Serra‐Medeiros, S. , Ortega, Z. , Antunes, P. C. , Miraglia Herrera, H. , & Oliveira‐Santos, L. G. R. (2021). Space use and activity of capybaras in an urban area. Journal of Mammalogy, 102(3), 814–825. 10.1093/jmammal/gyab005

[jane70040-bib-0163] Shaw, A. K. (2020). Causes and consequences of individual variation in animal movement. Movement Ecology, 8(1), 12. 10.1186/s40462-020-0197-x 32099656 PMC7027015

[jane70040-bib-0164] Shepard, E. L. C. , & Lambertucci, S. A. (2013). From daily movements to population distributions: Weather affects competitive ability in a guild of soaring birds. Journal of the Royal Society Interface, 10(88), 20130612. 10.1098/rsif.2013.0612 24026471 PMC3785828

[jane70040-bib-0165] Shepard, E. L. C. , Williamson, C. , & Windsor, S. P. (2016). Fine‐scale flight strategies of gulls in urban airflows indicate risk and reward in city living. Philosophical Transactions of the Royal Society, B: Biological Sciences, 371(1704), 20150394. 10.1098/rstb.2015.0394 PMC4992718 27528784

[jane70040-bib-0166] Shepard, E. L. C. , Wilson, R. P. , Rees, W. G. , Grundy, E. , Lambertucci, S. A. , & Vosper, S. B. (2013). Energy landscapes shape animal movement ecology. The American Naturalist, 182(3), 298–312. 10.1086/671257 23933722

[jane70040-bib-0167] Shochat, E. , Warren, P. S. , Faeth, S. H. , McIntyre, N. E. , & Hope, D. (2006). From patterns to emerging processes in mechanistic urban ecology. Trends in Ecology & Evolution, 21(4), 186–191. 10.1016/j.tree.2005.11.019 16701084

[jane70040-bib-0168] Signer, J. , Fieberg, J. , Reineking, B. , Schlägel, U. , Smith, B. , Balkenhol, N. , & Avgar, T. (2024). Simulating animal space use from fitted integrated step‐selection functions (iSSF). Methods in Ecology and Evolution, 15(1), 43–50. 10.1111/2041-210X.14263

[jane70040-bib-0169] Sih, A. (2013). Understanding variation in behavioural responses to human‐induced rapid environmental change: A conceptual overview. Animal Behaviour, 85(5), 1077–1088. 10.1016/j.anbehav.2013.02.017

[jane70040-bib-0170] Silovský, V. , Landler, L. , Faltusová, M. , Börger, L. , Burda, H. , Holton, M. , Lagner, O. , Malkemper, E. P. , Olejarz, A. , Spießberger, M. , Váchal, A. , & Ježek, M. (2024). A GPS assisted translocation experiment to study the homing behavior of red deer. Scientific Reports, 14(1), 6770. 10.1038/s41598-024-56951-0 38514686 PMC10958021

[jane70040-bib-0171] Simonis, A. E. , Brownell, R. L. , Thayre, B. J. , Trickey, J. S. , Oleson, E. M. , Huntington, R. , & Baumann‐Pickering, S. (2020). Co‐occurrence of beaked whale strandings and naval sonar in the Mariana Islands, Western Pacific. Proceedings of the Royal Society B: Biological Sciences, 287(1921), 20200070. 10.1098/rspb.2020.0070 PMC7062028 32070257

[jane70040-bib-0172] Skarin, A. , Nellemann, C. , Rönnegård, L. , Sandström, P. , & Lundqvist, H. (2015). Wind farm construction impacts reindeer migration and movement corridors. Landscape Ecology, 30(8), 1527–1540. 10.1007/s10980-015-0210-8

[jane70040-bib-0173] Soriano‐Redondo, A. , Bearhop, S. , Cleasby, I. R. , Lock, L. , Votier, S. C. , & Hilton, G. M. (2016). Ecological responses to extreme flooding events: A Case study with a reintroduced Bird. Scientific Reports, 6(1), 28595. 10.1038/srep28595 27345214 PMC4922006

[jane70040-bib-0174] Spelt, A. , Williamson, C. , Shamoun‐Baranes, J. , Shepard, E. , Rock, P. , & Windsor, S. (2019). Habitat use of urban‐nesting lesser black‐backed gulls during the breeding season. Scientific Reports, 9(1), 10527. 10.1038/s41598-019-46890-6 31324838 PMC6642139

[jane70040-bib-0175] Strum, S. C. (2010). The development of primate raiding: Implications for management and conservation. International Journal of Primatology, 31(1), 133–156. 10.1007/s10764-009-9387-5 PMC2819593 20174437

[jane70040-bib-0176] Sueur, C. (2023). A deep learning model to unlock secrets of animal movement and behaviour. Peer community in Ecology, 1, 100531. 10.24072/pci.ecology.100531

[jane70040-bib-0177] Sunday, J. M. , Bates, A. E. , & Dulvy, N. K. (2012). Thermal tolerance and the global redistribution of animals. Nature Climate Change, 2(9), 686–690. 10.1038/nclimate1539

[jane70040-bib-0178] Suraci, J. P. , Clinchy, M. , Zanette, L. Y. , & Wilmers, C. C. (2019). Fear of humans as apex predators has landscape‐scale impacts from mountain lions to mice. Ecology Letters, 22(10), 1578–1586. 10.1111/ele.13344 31313436

[jane70040-bib-0179] Svenning, J.‐C. , Buitenwerf, R. , & Le Roux, E. (2024). Trophic rewilding as a restoration approach under emerging novel biosphere conditions. Current Biology, 34(9), R435–R451. 10.1016/j.cub.2024.02.044 38714176

[jane70040-bib-0180] Svenning, J.‐C. , McGeoch, M. A. , Normand, S. , Ordonez, A. , & Riede, F. (2024). Navigating ecological novelty towards planetary stewardship: Challenges and opportunities in biodiversity dynamics in a transforming biosphere. Philosophical Transactions of the Royal Society, B: Biological Sciences, 379(1902), 20230008. 10.1098/rstb.2023.0008 PMC10999270 38583480

[jane70040-bib-0181] Symons, S. C. , & Diamond, A. W. (2019). Short‐term tracking tag attachment disrupts chick provisioning by Atlantic puffins *Fratercula arctica* and razorbills *Alca torda* . Bird Study, 66(1), 53–63. 10.1080/00063657.2019.1612850

[jane70040-bib-0182] te Velde, K. , Mairo, A. , Peeters, E. T. H. M. , Winter, H. V. , Tudorache, C. , & Slabbekoorn, H. (2024). Natural soundscapes of lowland river habitats and the potential threat of urban noise pollution to migratory fish. Environmental Pollution, 359, 124517. 10.1016/j.envpol.2024.124517 39002749

[jane70040-bib-0183] Trombulak, S. C. , & Frissell, C. A. (2000). Review of ecological effects of roads on terrestrial and aquatic communities. Conservation Biology, 14(1), 18–30. 10.1046/j.1523-1739.2000.99084.x

[jane70040-bib-0184] Tucker, M. A. , Böhning‐Gaese, K. , Fagan, W. F. , Fryxell, J. M. , Van Moorter, B. , Alberts, S. C. , Ali, A. H. , Allen, A. M. , Attias, N. , Avgar, T. , Bartlam‐Brooks, H. , Bayarbaatar, B. , Belant, J. L. , Bertassoni, A. , Beyer, D. , Bidner, L. , van Beest, F. M. , Blake, S. , Blaum, N. , … Mueller, T. (2018). Moving in the Anthropocene: Global reductions in terrestrial mammalian movements. Science, 359(6374), 466–469. 10.1126/science.aam9712 29371471

[jane70040-bib-0185] Tucker, M. A. , Schipper, A. M. , Adams, T. S. F. , Attias, N. , Avgar, T. , Babic, N. L. , Barker, K. J. , Bastille‐Rousseau, G. , Behr, D. M. , Belant, J. L. , Beyer, D. E. , Blaum, N. , Blount, J. D. , Bockmühl, D. , Pires Boulhosa, R. L. , Brown, M. B. , Buuveibaatar, B. , Cagnacci, F. , Calabrese, J. M. , … Mueller, T. (2023). Behavioral responses of terrestrial mammals to COVID‐19 lockdowns. Science, 380(6649), 1059–1064. 10.1126/science.abo6499 37289888

[jane70040-bib-0186] Tuia, D. , Kellenberger, B. , Beery, S. , Costelloe, B. R. , Zuffi, S. , Risse, B. , Mathis, A. , Mathis, M. W. , van Langevelde, F. , Burghardt, T. , Kays, R. , Klinck, H. , Wikelski, M. , Couzin, I. D. , van Horn, G. , Crofoot, M. C. , Stewart, C. V. , & Berger‐Wolf, T. (2022). Perspectives in machine learning for wildlife conservation. Nature Communications, 13(1), 792. 10.1038/s41467-022-27980-y PMC8828720 35140206

[jane70040-bib-0187] Tuomainen, U. , & Candolin, U. (2011). Behavioural responses to human‐induced environmental change. Biological Reviews, 86(3), 640–657. 10.1111/j.1469-185X.2010.00164.x 20977599

[jane70040-bib-0188] Tuxbury, S. M. , & Salmon, M. (2005). Competitive interactions between artificial lighting and natural cues during seafinding by hatchling marine turtles. Biological Conservation, 121(2), 311–316. 10.1016/j.biocon.2004.04.022

[jane70040-bib-0189] Uyeda, L. T. , Iskandar, E. , Kyes, R. C. , & Wirsing, A. J. (2015). Encounter rates, agonistic interactions, and social hierarchy among garbage‐feeding water monitor lizards (*Varanus salvator bivittatus*) on Tinjil Island, Indonesia. Herpetological Conservation and Biology, 10(2), 753–764.

[jane70040-bib-0190] van der Ree, R. , Smith, D. J. , & Grilo, C. (2015). The ecological effects of linear infrastructure and traffic. In R. T. T. Forman (Ed.), Handbook of road ecology (pp. 1–9). John Wiley & Sons, Ltd. 10.1002/9781118568170.ch1

[jane70040-bib-0212] van Klink, R. , August, T. , Bas, Y. , Bodesheim, P. , Bonn, A. , Fossøy, F. , Høye, T. T. , Jongejans, E. , Menz, M. H. M. , Miraldo, A. , Roslin, T. , Roy, H. E. , Ruczyński, I. , Schigel, D. , Schäffler, L. , Sheard, J. K. , Svenningsen, C. , Tschan, G. F. , Wäldchen, J. , … Bowler, D. E. (2022). Emerging technologies revolutionise insect ecology and monitoring. Trends in Ecology & Evolution, 37(10), 872–885. 10.1016/j.tree.2022.06.001 35811172

[jane70040-bib-0191] Vedor, M. , Queiroz, N. , Mucientes, G. , Couto, A. , da Costa, I. , dos Santos, A. , Vandeperre, F. , Fontes, J. , Afonso, P. , Rosa, R. , Humphries, N. E. , & Sims, D. W. (2021). Climate‐driven deoxygenation elevates fishing vulnerability for the ocean's widest ranging shark. eLife, 10, e62508. 10.7554/eLife.62508 33461659 PMC7815312

[jane70040-bib-0192] Venter, O. , Sanderson, E. W. , Magrach, A. , Allan, J. R. , Beher, J. , Jones, K. R. , Possingham, H. P. , Laurance, W. F. , Wood, P. , Fekete, B. M. , Levy, M. A. , & Watson, J. E. M. (2016). Sixteen years of change in the global terrestrial human footprint and implications for biodiversity conservation. Nature Communications, 7(1), 12558. 10.1038/ncomms12558 PMC4996975 27552116

[jane70040-bib-0193] Vilà, M. , Espinar, J. L. , Hejda, M. , Hulme, P. E. , Jarošík, V. , Maron, J. L. , Pergl, J. , Schaffner, U. , Sun, Y. , & Pyšek, P. (2011). Ecological impacts of invasive alien plants: A meta‐analysis of their effects on species, communities and ecosystems. Ecology Letters, 14(7), 702–708. 10.1111/j.1461-0248.2011.01628.x 21592274

[jane70040-bib-0194] Wacher, T. , Amin, R. , Newby, J. , Hatcha, M. H. , Abeye, K. , Ali, H. , Bourtchiakbé, S. Z. , & Banlongar, F. N. (2023). Gazelle–livestock interactions and impact of water resource development in the Ouadi Rimé–Ouadi Achim reserve, Chad. Oryx, 57(2), 205–215. 10.1017/S0030605321001629

[jane70040-bib-0195] Webster, M. M. , & Rutz, C. (2020). How STRANGE are your study animals? Nature, 582(7812), 337–340. 10.1038/d41586-020-01751-5 32541916

[jane70040-bib-0196] Werner, K. M. , Haslob, H. , Reichel, A. F. , Gimpel, A. , & Stelzenmüller, V. (2024). Offshore wind farm foundations as artificial reefs: The devil is in the detail. Fisheries Research, 272, 106937. 10.1016/j.fishres.2024.106937

[jane70040-bib-0197] Whyte, K. F. , Russell, D. J. F. , Sparling, C. E. , Binnerts, B. , & Hastie, G. D. (2020). Estimating the effects of pile driving sounds on seals: Pitfalls and possibilitiesa. The Journal of the Acoustical Society of America, 147(6), 3948–3958. 10.1121/10.0001408 32611185

[jane70040-bib-0198] Wijeyakulasuriya, D. A. , Eisenhauer, E. W. , Shaby, B. A. , & Hanks, E. M. (2020). Machine learning for modeling animal movement. PLoS One, 15(7), e0235750. 10.1371/journal.pone.0235750 32716917 PMC7384613

[jane70040-bib-0199] Williams, B. K. , & Brown, E. D. (2024). Managing ecosystems with resist–accept–direct (RAD). Methods in Ecology and Evolution, 15(5), 796–805. 10.1111/2041-210X.14309

[jane70040-bib-0200] Williams, H. J. , Taylor, L. A. , Benhamou, S. , Bijleveld, A. I. , Clay, T. A. , de Grissac, S. , Demšar, U. , English, H. M. , Franconi, N. , Gómez‐Laich, A. , Griffiths, R. C. , Kay, W. P. , Morales, J. M. , Potts, J. R. , Rogerson, K. F. , Rutz, C. , Spelt, A. , Trevail, A. M. , Wilson, R. P. , & Börger, L. (2020). Optimizing the use of biologgers for movement ecology research. The Journal of Animal Ecology, 89(1), 186–206. 10.1111/1365-2656.13094 31424571 PMC7041970

[jane70040-bib-0201] Williamson, M. J. , Tebbs, E. J. , Curnick, D. J. , Ferretti, F. , Carlisle, A. B. , Chapple, T. K. , Schallert, R. J. , Tickler, D. M. , Block, B. A. , & Jacoby, D. M. P. (2024). Environmental stress reduces shark residency to coral reefs. Communications Biology, 7(1), 1–12. 10.1038/s42003-024-06707-3 39251811 PMC11385207

[jane70040-bib-0202] Yanco, S. W. , Rutz, C. , Abrahms, B. , Cooper, N. W. , Marra, P. P. , Mueller, T. , Weeks, B. C. , Wikelski, M. , & Oliver, R. Y. (2025). Tracking individual animals can reveal the mechanisms of species loss. Trends in Ecology & Evolution, 40(1), 47–56. 10.1016/j.tree.2024.09.008 39505577

[jane70040-bib-0203] Yates, K. L. , Bouchet, P. J. , Caley, M. J. , Mengersen, K. , Randin, C. F. , Parnell, S. , Fielding, A. H. , Bamford, A. J. , Ban, S. , Barbosa, A. M. , Dormann, C. F. , Elith, J. , Embling, C. B. , Ervin, G. N. , Fisher, R. , Gould, S. , Graf, R. F. , Gregr, E. J. , Halpin, P. N. , … Sequeira, A. M. M. (2018). Outstanding challenges in the transferability of ecological models. Trends in Ecology & Evolution, 33(10), 790–802. 10.1016/j.tree.2018.08.001 30166069

[jane70040-bib-0204] Yirga, G. , Leirs, H. , Iongh, H. H. D. , Asmelash, T. , Gebrehiwot, K. , Deckers, J. , & Bauer, H. (2015). Spotted hyena (*Crocuta crocuta*) concentrate around urban waste dumps across Tigray, northern Ethiopia. Wildlife Research, 42(7), 563–569. 10.1071/WR14228

[jane70040-bib-0205] Yun, J. , Shin, W. , Kim, J. , Thorne, J. H. , & Song, Y. (2024). Citizen‐science data identifies the daily movement patterns and habitat associations of a nocturnal urban‐invading bird species (*Corvus frugilegus*). Urban Ecosystems, 27(5), 1407–1416. 10.1007/s11252-024-01508-2

[jane70040-bib-0206] Zanette, L. Y. , Frizzelle, N. R. , Clinchy, M. , Peel, M. J. S. , Keller, C. B. , Huebner, S. E. , & Packer, C. (2023). Fear of the human “super predator” pervades the south African savanna. Current Biology, 33(21), 4689–4696.e4. 10.1016/j.cub.2023.08.089 37802052

[jane70040-bib-0207] Zeller, K. A. , Ditmer, M. A. , Squires, J. R. , Rice, W. L. , Wilder, J. , DeLong, D. , Egan, A. , Pennington, N. , Wang, C. A. , Plucinski, J. , & Barber, J. R. (2024). Experimental recreationist noise alters behavior and space use of wildlife. Current Biology, 34(13), 2997–3004.e3. 10.1016/j.cub.2024.05.030 38876101

